# Regenerative Nano-scaffolds for Tissue Engineering: From Bench to Clinical Translation

**DOI:** 10.34133/bmr.0414

**Published:** 2026-08-31

**Authors:** Woochan Kim, Sangbae Park, Sang Won Beom, Selin Choi, Hye-Won Yang, Dream Kim, Shinyull Lee, Harshita Sharma, Chaeyeon Park, Won-Pyo Lee, Jangho Kim

**Affiliations:** ^1^ Department of Convergence Biosystems Engineering, Chonnam National University, Gwangju 61186, Republic of Korea.; ^2^ Department of Rural and Biosystems Engineering, Chonnam National University, Gwangju 61186, Republic of Korea.; ^3^ Interdisciplinary Program in IT-Bio Convergence System, Chonnam National University, Gwangju 61186, Republic of Korea.; ^4^ Department of Biosystems Engineering, Seoul National University, Seoul 08826, Republic of Korea.; ^5^ Integrated Major in Global Smart Farm, College of Agriculture and Life Sciences, Seoul National University, Seoul 08826, Republic of Korea.; ^6^ Research Institute for Agriculture and Life Sciences, Seoul National University, Seoul 08826, Republic of Korea.; ^7^ Institute of Nano-Stem Cells Therapeutics, NANOBIOSYSTEM Co., Ltd., Gwangju 61003, Republic of Korea.; ^8^ Department of Periodontology, School of Dentistry, Chosun University, Gwangju 61452, Republic of Korea.

## Abstract

Biomedical membranes are among the most widely used biomaterials in clinical regenerative medicine due to their ease of application, biocompatibility, and versatility across multiple tissues. However, conventional membranes have been limited to passive roles serving as physical barriers or wound coverings without an intrinsic capability to initiate or orchestrate true tissue regeneration. Here, we report the design, development, and clinical validation of an active tissue-regenerative biomedical membrane patch, aiming to advance biomedical membranes from passive protection toward active human tissue regeneration. In this study, we developed a multifunctional, collagen-coated polylactic-*co*-glycolic acid nanotopographical scaffold (Col-NS) that mimics the native extracellular matrix to enhance soft- and hard-tissue regeneration. In a clinical trial for laser-induced human skin injury, Col-NS substantially improved healing outcomes, achieving accelerated wound contraction, dermal volume restoration, reduced surface roughness, and decreased transepidermal water loss relative to standard care. In human dental procedures, including alveolar ridge preservation and guided bone regeneration, Col-NS enabled robust bone formation, stable implant osseointegration, and complication-free recovery. These findings demonstrate the translational feasibility of an extracellular-matrix-mimetic nanoengineered scaffold across soft- and hard-tissue applications and support its potential as a clinically relevant platform for regenerative medicine. This work may contribute to broadening the clinical role of biomedical patches from passive coverings toward active regenerative platforms.

## Introduction

Membrane-based biomedical scaffolds are among the most widely used medical devices for tissue regeneration in clinical applications [1]. Although membrane-based scaffolds are widely used, most of them serve only passive roles such as wound coverage and barrier protection, and they lack intrinsic therapeutic functionality, including the ability to actively promote tissue regeneration [2,3]. The ideal strategy for the restoration of damaged human tissues comprises scaffolds that provide structural support for tissue growth, cells capable of generating the desired tissue, and biochemical or environmental cues that regulate cell growth and phenotype [4]. However, the biomedical scaffolds currently used in real clinical settings mainly function as passive structural support rather than as active participants in the regenerative process [5]. Most commercially available membranes or scaffolds are designed to act as physical barriers to prevent infection or undesired tissue invasion, but they fail to deliver biochemical or topographical signals that can actively guide cellular behavior and enhance regenerative outcomes. As a result, their clinical effectiveness often depends on the addition of external biological agents, such as growth factors, stem cells, or drugs, rather than on the scaffold’s intrinsic functionality. To overcome these limitations, recent efforts have focused on developing bioactive or multifunctional scaffolds capable of mimicking the extracellular matrix (ECM) and providing physicochemical cues that can modulate cell adhesion, proliferation, and differentiation [2,6]. Such next-generation scaffolds aim not only to support tissue growth mechanically but also to orchestrate biological processes at the molecular and cellular levels, ultimately enabling more effective and predictable tissue regeneration in clinical applications.

Collagen-based membrane biomedical scaffolds are widely recognized as promising biomaterials for tissue regeneration, due to their inherent biocompatibility, biodegradability, low immunogenicity, and essential role as a primary structural component of the ECM [7]. These devices support key cellular activities, such as adhesion, migration, and proliferation, which are critical for effective tissue regeneration [8,9]. Clinically, collagen is extensively utilized to regenerate various tissues, including bone, cartilage, skin, nerve, and periodontal tissues, owing to its favorable biological profile and its exceptional capacity to preserve structural integrity during early tissue formation [10,11]. However, despite these advantages, collagen-based devices face substantial limitations. A major challenge is their susceptibility to enzymatic degradation, which can lead to accelerated degradation in vivo [12]. This rapid degradation can compromise scaffold integrity before adequate tissue regeneration occurs, resulting in suboptimal tissue formation that falls short of native tissue quality. Furthermore, collagen possesses inherently poor mechanical properties, including low tensile strength and limited resistance to deformation, rendering it unsuitable for applications such as bone regeneration in weight-bearing areas [13]. In skin regeneration, collagen-based devices often lack the mechanical stability needed to maintain structural integrity over time, which can hinder effective re-epithelialization and dermal tissue remodeling. The high costs associated with extracting and purifying medical-grade collagen also limit its scalability and overall cost-effectiveness [14]. These inherent challenges underscore the necessity for advanced strategies to enhance the clinical performance of collagen-based biomaterials.

Biodegradable polymers, such as polylactic acid, polyglycolic acid, and polylactic-*co*-glycolic acid (PLGA), are widely used in temporary implantable medical devices, where they degrade in the body through various mechanisms after implantation [15,16]. These materials offer a promising alternative to overcome several limitations associated with collagen-based medical devices. They exhibit favorable mechanical strength, tunable degradation rates, and excellent processability, making them suitable for diverse biomedical applications [17]. They can also be precisely engineered to match the mechanical, biochemical, and structural requirements of target tissues through advanced microfabrication and nanofabrication techniques [18]. As temporary scaffolds, these polymers facilitate tissue remodeling by providing physical support and biochemical cues essential for effective regeneration [19]. Once tissue repair is complete, they are naturally absorbed by the body, thus eliminating the necessity for surgical removal [20]. However, major limitations include poor cell affinity, undesirable inflammatory responses, and the accumulation of acidic degradation by-products, which may impair tissue healing and lead to fibrosis or other complications [21]. These challenges highlight the need for the development of biodegradable medical devices to effectively promote tissue regeneration and address these clinical limitations.

Tissue engineering and regenerative medicine have emerged as transformative fields capable of addressing the growing demand for organ and tissue repair. A key element of these advancements is the development of scaffolds that not only facilitate cellular adhesion but also replicate the structural and biochemical complexity of the native ECM to guide tissue regeneration [22]. Recent scaffold engineering efforts have focused on recapitulating the nanostructural and compositional features of the ECM to enhance scaffold functionality [23]. Strategies such as collagen coating and biomimetic nanostructure design are particularly promising [24]. These approaches aim to bridge the gap between synthetic materials and the natural cellular environment by creating a more physiologically relevant microenvironment for regeneration [25,26]. Collagen coatings enhance cell adhesion and act as reservoirs for bioactive molecules, while ECM-mimetic nanostructures introduce topographical cues that closely resemble the native tissue architecture, thereby promoting favorable cell–material interactions [27]. The integration of these design strategies holds substantial promise for improving regeneration across a range of tissues, from skin and bone to more complex organs. Moreover, the precise control of nanoscale features influences stem cell behavior, directing their fate toward specific lineages, which is critical for targeted tissue regeneration [28]. Despite these advances, achieving scalable, cost-effective, and clinically translatable solutions remains challenging.

In this study, we developed and translationally evaluated a collagen-coated PLGA nanotopographical membrane designed to provide complementary biophysical and biochemical cues for tissue regeneration. The principal contribution of this work lies not in the individual use of PLGA, collagen, or nanotopography but in their integrated implementation and multilevel validation across mechanistic in vitro studies, normal and diabetic wound models, a calvarial defect model, and human skin and alveolar bone applications. Therefore, this study aims to develop and comprehensively validate a collagen-coated PLGA nanotopographical scaffold (Col-NS) that mimics the biochemical and nanostructural features of the ECM, with a primary focus on its clinical applicability in skin and alveolar bone regeneration. Col-NS was fabricated by coating aligned nanotopographical structures of biodegradable PLGA with type I collagen, aiming to enhance essential cellular behaviors such as adhesion, migration, proliferation, and differentiation, which are required for effective tissue regeneration. In the preclinical stage, Col-NS was evaluated in normal and diabetic wound models, as well as in a cranial bone defect model. In the clinical stage, Col-NS was applied in human case studies, including patients undergoing skin wound repair and alveolar bone regeneration during dental implant procedures, to assess its clinical safety, handling characteristics, and therapeutic performance. The findings could underscore the clinical safety and therapeutic potential of Col-NS as a next-generation platform for translational tissue regeneration.

## Materials and Methods

### Design and fabrication of the scaffolds

A droplet of ultraviolet (UV)-curable polyurethane acrylate (PUA) precursor solution containing a photoinitiator (Changsung Sheet, Korea) was dispensed onto a silicon master mold featuring nanoscale linear grooves and ridges (800 nm) fabricated using conventional photolithography and reactive ion etching. A poly(ethylene terephthalate) film was then uniformly applied over the mold, driven by capillary forces. Following this, the mold was exposed to UV light (*λ* = 352 nm, 40 W) for 60 s to cure the PUA. After polymerization, the resulting nanotopographic PUA replica was carefully detached from the master mold using tweezers and subjected to overnight UV exposure to eliminate residual acrylate groups. A 10% (w/v) PLGA solution in chloroform (300 μl) was dispensed onto a nanotopographical PUA replica. A flat polydimethylsiloxane (PDMS) top block was then placed on top to form a nanotopographical PLGA film layer, and the solvent was allowed to evaporate. Afterward, the PDMS block was removed, and the sample was preheated on a hot plate to ensure complete solvent removal. The sample was then cooled to room temperature, and the nanotopographical PUA mold was peeled off, resulting in a nanotopographical PLGA scaffold with ridge and groove widths of 800 nm.

### Characterization of the scaffolds

FS, NS, and Col-NS were analyzed using a high-resolution field-emission scanning electron microscope. The surface morphology of all scaffold types fabricated in this study was imaged using a Zeiss Gemini SEM 500 (Zeiss Ltd., Germany) at an acceleration voltage of 15.0 kV and an average working distance of 8.8 mm, following platinum coating.

### Mechanical properties of the scaffolds

Mechanical properties (i.e., stress and strain) were analyzed using an MCT-1150 tensile testing system (A&D Company, Japan). Rectangular patches (width 12 mm and length 20 mm) were tested at a crosshead speed of 100 mm/min, with tensile load applied along the direction of aligned nanotopography. The sample was measured at a 10-mm gauge length. Adhesion stress testing was performed at a crosshead speed of 1.5 mm/s, and the peak load, along with the displacement at the detachment point, was measured and documented.

### Scanning electron microscope imaging of cells

NIH 3T3 cell-seeded scaffolds were fixed in 1× phosphate-buffered saline (PBS; Sigma-Aldrich, MO, USA) containing 2% paraformaldehyde and 2% glutaraldehyde (Sigma-Aldrich, MO, USA) for 4 h. After fixation, the scaffolds were washed 3 times with 1× PBS, postfixed with 1% osmium tetroxide (Sigma-Aldrich, MO, USA) for 1.5 h, and washed again 3 times with 1× PBS. Dehydration was performed using a graded ethanol series (30%, 50%, 70%, 80%, 90%, and 100% v/v). Finally, samples were coated with a Pt layer (~5-nm thickness) via metal sputtering and imaged using Zeiss Gemini SEM 500 (Zeiss Ltd., Germany).

### Immunocytochemistry

NIH 3T3 cells were used for the in vitro study and cultured for 24 h on collagen-coated nanotopographical scaffolds at concentrations of 0.1%, 0.5%, 1%, and 2%. Following incubation, the attached cells were fixed with 4% paraformaldehyde (Sigma-Aldrich, MO, USA) for 5 min and washed 3 times with 1 × PBS. Fixed cells were permeabilized with 0.1% Triton X-100 in 1 × PBS for 10 min and washed again 3 times with 1 × PBS. Cells were then blocked with 1% bovine serum albumin for 1 h and incubated overnight at 4 °C with bovine serum albumin-diluted anti-vinculin primary antibody (Sigma-Aldrich, MO, USA). After 3 additional PBS washes, the cells were incubated with PBS-diluted fluorescein isothiocyanate- and tetramethylrhodamine-conjugated phalloidin and anti-vinculin (Sigma-Aldrich, MO, USA). Nuclei were then counterstained with diluted 4′,6-diamidino-2-phenylindole (Sigma-Aldrich, MO, USA). Fluorescently labeled cells were imaged using a laser confocal scanning microscope (Zeiss LSM 980 with Airyscan 2; Zeiss, Germany).

### Cell morphology analysis

To evaluate the effect of collagen coating concentration on fibroblast morphology and alignment, NIH 3T3 cells cultured for 24 h on nanotopographical scaffolds coated with 0.1%, 0.5%, 1%, and 2% collagen were imaged using a laser confocal scanning microscope (Zeiss LSM 980 with Airyscan 2; Zeiss, Germany) and analyzed with ImageJ (National Institutes of Health [NIH], Bethesda, MD, USA). For each cell, a best-fit ellipse was applied to the F-actin- or vinculin-defined cell boundary to extract the major and minor axis lengths and the orientation angle. The cell aspect ratio was calculated as the major-to-minor axis ratio, and the cell elongation index as (major − minor)/(major + minor), ranging from 0 (circular) to 1 (highly elongated). Cell orientation was defined as the angle between the major axis and the underlying nanotopography (0° = parallel alignment) and visualized as polar histograms spanning −90° to 90°. In addition, individual vinculin-positive focal adhesions were segmented by intensity thresholding, and the focal adhesion area was quantified as the mean area of the segmented puncta per cell. A minimum of 30 cells from at least 3 independent samples were analyzed per condition, and all data are presented as mean ± standard deviation.

### Clinical case study: Human skin tissue regeneration

The clinical trial was conducted under the supervision of the Clinical Trial Center at SkinMed Co., Ltd., in accordance with the Guidelines for Human Application Tests and Efficacy Tests of Cosmetics (Guideline No. 0333-02) and the Guidelines for Test Methods for Substantiating Cosmetic Labeling and Advertising Claims (Guideline No. 0353-02), issued by the Ministry of Food and Drug Safety, Republic of Korea. The study also adhered to the standard operating procedures of the SkinMed Clinical Trial Center. This clinical study on human skin tissue regeneration aimed to evaluate the efficacy of collagen-coated nanotopographical scaffolds in improving skin surface roughness and tissue recovery following laser-induced injury. Overall, 25 healthy adult participants, aged 20 to 60 years, were enrolled in the study. The intervention was applied to the facial region, specifically to laser-treated pigmented lesions. Participants were assigned to 1 of 3 treatment groups: (a) collagen-coated nanotopographical scaffolds applied once daily for 7 d, (b) the same scaffold regimen combined with a regenerative cream applied from day 4 to day 7, and (c) a control group treated with Duoderm patches once daily for 7 d. Evaluation time points included baseline (before product application), immediately after laser treatment, 3 d postapplication, and 7 d postapplication. Skin condition was assessed using 4 different parameters. First, the wound area was evaluated in pigmentation mode using the Antera 3D imaging system (Miravex, Ireland). Images were analyzed with the Image-Pro software (Media Cybernetics, MD, USA), and a reduction in area (A.U.) was interpreted as improved wound contraction. Second, skin depression volume was measured using the Antera 3D system and its dedicated analysis software. This quantified surface depressions at the lesion site, with lower values (mm^3^) indicating improvement in tissue restoration and smoother skin contours. Third, skin surface roughness was assessed using the Visioscan VC 20plus device (Courage + Khazaka electronic GmbH, Germany), which captures high-resolution topographic images using a video sensor and reflected light. The average roughness value (R3) was utilized as a quantitative index; a decrease in R3 (A.U.) indicated a reduced roughness and enhanced skin texture. Lastly, transepidermal water loss (TEWL) was measured to evaluate skin barrier function using Tewameter TM 300 (Courage + Khazaka electronic GmbH, Germany). This instrument measures water evaporation through the skin using paired sensors embedded in a cylindrical probe. Higher TEWL values reflect barrier disruption, whereas decreased values (g/m^2^ h) indicate barrier recovery.

### Clinical case study: Human alveolar bone regeneration

This clinical study enrolled patients from the Department of Periodontology at Chosun University Dental Hospital who underwent implant-related procedures involving alveolar ridge preservation (ARP) or guided bone regeneration (GBR) with the Col-NS membrane. Inclusion criteria required patients to have completed prosthetic loading following surgery and to possess sufficient clinical photographs and radiographs for analysis. Patients without follow-up data or insufficient documentation were excluded. Ten patients met the inclusion criteria, with ages ranging from 46 to 67 years (Table 1). All participants were fully informed of the treatment procedures and provided written consent prior to surgery. The study protocol was approved by the Institutional Review Board of Chosun University Dental Hospital, Gwangju, Korea (IRB No. CUDHIRB-2307-004).

**Table 1.** Characteristics of the patients

Case	Sex	Age	PMH	Procedure	Location	Implant prognosis	Complications
1	F	50	HTN	ARP, GBR	#17, #15	Success	None
2	F	65	HTN, CVD	GBR	#26	Success	None
3	F	67	OP	ARP	#26	Success	None
4	F	65	CVD, OP	ARP	#45	Success	None
5	M	66	CVD	GBR	#26, #27	Success	None
6	M	46	None	GBR	#16	Success	None
7	M	66	CVD	GBR	#47, #46, #35, #37	Success	None
8	M	65	DM	ARP	#36	Success	None
9	M	53	None	ARP	#45, #43	Success	None
10	M	48	CVD	GBR	#17, #15	Success	None

PMH, past medical history; F, female; M, male; HTN, hypertension; ARP, alveolar ridge preservation; GBR, guided bone regeneration; CVD, cardiovascular disease; OP, osteoporosis; DM, diabetes mellitus

### In vivo study on skin tissue regeneration

All animal experiments were approved by the Institutional Animal Care and Use Committee of Chonnam National University. The dorsal hair of adult Sprague-Dawley rats (200 to 300 g) and Zucker Diabetic-Sprague-Dawley rats (500 g) was shaved under inhalation anesthesia with isoflurane; 8-mm-diameter full-thickness skin defects were then created, and prefabricated scaffolds (10 mm in diameter) were placed over the wounds, followed by monitoring of skin regeneration. Wound areas were measured using the ImageJ software (NIH, Bethesda, MD, USA), and wound closure was calculated as follows: Wound ratio (%) = [(S0 − S1)/S0] × 100, where S0 is the initial wound area and S1 is the area at each time point. Wound tissues were fixed in 10% neutral-buffered formalin for 24 h prior to further histological processing. The tissues were then dehydrated, paraffin-embedded, and sectioned into 4-μm-thick slices. The sections were deparaffinized and rehydrated using xylene and a graded ethanol series. The slides were stained with Harris hematoxylin for 5 min and eosin for 1 min, followed by Masson’s trichrome (MT) before imaging. The hematoxylin and eosin (H&E)-stained and MT-stained slides were examined under a bright-field optical microscope. The ImageJ software was used to quantify epidermal thickness, dermal thickness, collagen fibers, and hair follicle counts.

### In vivo study on bone tissue regeneration

All animal procedures were approved by Chonnam National University Hospital. Six-week-old male C57BL/6 mice were randomly assigned to 2 groups (*n* = 5 per group): defect (control) and Col-NS. The mice were fully anesthetized via intraperitoneal injection of Zoletil (0.006 cm^3^/10 g) and Rompun (0.004 cm^3^/10 g). The scalps were shaved and disinfected, and a midline skin incision approximately 3.0 cm in length was made over the calvaria to expose the skull. A unilateral circular bone defect (diameter: 5 mm) was created on the exposed calvarial surface using an electric drill. Prepared scaffolds (diameter: 7 mm) were then placed over the defects, and the skin was sutured. Following surgery, the animals were allowed to recover under warm ambient conditions. The mice were euthanized at 8 weeks postoperatively, and tissues, including the calvarial bone and defect area, were harvested. Micro-computed tomography (μ-CT) of the calvarial bones was performed using a Skyscan 1172 system (Skyscan, Kontich, Belgium) at a resolution of 11.38 μm per pixel and an exposure time of 316 ms, with the x-ray source set to 80 kV and 124 μA. On average, 488 slices per sample were acquired. μ-CT images were analyzed using the MIMICS 14.0 3-dimensional (3D) imaging software (Materialise, Leuven, Belgium). Harvested bones were fixed in 10% formalin and decalcified in 0.5 M ethylenediaminetetraacetic acid (pH 7.4) at room temperature for 7 d. Samples were paraffin-embedded, sectioned at a thickness of 5 μm, and stained with H&E and MT. Histological images were captured using the Aperio ImageScope software (Leica, CA, USA).

### Cell migration assay

To evaluate the migration and guidance behavior of NIH 3T3 cells on tissue culture polystyrene (TCPS), NS, and Col-NS, a thin PDMS barrier slab (diameter: 1 mm) was placed to create a cell-free region. Cells (1 × 10^6^ cells/sample) were seeded onto the scaffolds, and after attachment, the PDMS slab was manually removed using fine tweezers. Cell migration into the cleared region was monitored at 0, 12, 24, 48, and 60 h using an optical microscope (Zeiss, Oberkochen, Germany). The area covered by the migrating cells was analyzed using the ImageJ software.

### Cell attachment and viability analyses

To evaluate NIH 3T3 cell attachment and viability on the various scaffold samples, cells (1 × 10^4^ per sample) were cultured in standard Dulbecco’s modified Eagle’s medium (Sigma-Aldrich, MO, USA) containing 10% fetal bovine serum (Sigma-Aldrich, MO, USA) and 1% antibiotics (Sigma-Aldrich, MO, USA) under humidified conditions at 37 °C for 1, 3, and 5 d. Cell viability was quantified using the WST-1 assay (PreMix WST-1 Cell Proliferation Assay System, TaKaRa, Kusatsu, Japan), and absorbance was measured at 450 nm using a microplate spectrophotometer (iMark Microplate Absorbance Reader, Bio-Rad Laboratories, Hercules, CA, USA).

### Osteogenic differentiation and mineralization analyses

MG-63 human osteoblast-like cells were used to evaluate the osteogenic potential of the scaffolds. Cells were seeded onto TCPS, nanotopographical scaffolds (NS), and collagen-coated nanotopographical scaffolds (Col-NS) at a density of 1 × 10^4^ cells/sample and cultured at 37 °C in a humidified atmosphere containing 5% CO2. After cell attachment, the culture medium was replaced with osteogenic differentiation medium consisting of normal culture medium supplemented with 100 nM dexamethasone, 50 μM ascorbic acid, and 10 mM glycerol 2-phosphate. The cells were maintained for 7 or 14 d, with the medium replaced every 3 d. After 7 d of osteogenic induction, early osteogenic differentiation was evaluated by alkaline phosphatase (ALP) staining. The cells were washed with PBS, fixed with 4% paraformaldehyde, and subjected to ALP staining. ALP activity was further quantified using the SensoLyte pNPP Alkaline Phosphatase Assay Kit (AnaSpec) according to the manufacturer’s instructions. The absorbance of the extracted solutions was measured at 405 nm using iMark Microplate Absorbance Reader (Bio-Rad Laboratories, Hercules, CA, USA). After 14 d of osteogenic induction, extracellular calcium deposition and matrix mineralization were evaluated by Alizarin Red S staining (Sigma-Aldrich, MO, USA). Following staining, the samples were thoroughly washed to remove excess dye and imaged using an optical microscope. For quantitative analysis, the bound Alizarin Red S dye was extracted by destaining with cetylpyridinium chloride (Sigma-Aldrich, MO, USA), and the absorbance of the extracted solutions was measured at 570 nm using iMark Microplate Absorbance Reader (Bio-Rad Laboratories, Hercules, CA, USA).

### Western blot analysis

To evaluate the protein expression levels related to the transforming growth factor-β (TGF-β) and Hippo signaling pathways, NIH 3T3 cells were cultured on each scaffold for 24 h. The cells were then washed twice with cold PBS and lysed in RIPA buffer via sonication at 4 °C for 30 min. The resulting lysates were centrifuged at 12,000×g for 15 min at 4 °C, and the supernatants were collected. After an additional PBS wash, the cells were lysed again in a modified RIPA buffer (150 mM sodium chloride, 1% Triton X-100, 0.5% sodium deoxycholate, 0.1% sodium dodecyl sulfate [SDS], 50 mM Tris [pH 8.0], 1 mM phenylmethylsulfonyl fluoride, 2 μg/ml leupeptin, 1 μg/ml pepstatin, 1 mM sodium orthovanadate, and 100 mM sodium fluoride) at 4 °C for 30 min. The lysates were purified through centrifugation at 13,000×g for 30 min at 4 °C. The protein concentrations of the cell lysates were determined using the Pierce Rapid Gold BCA Protein Assay Kit (Pierce, Rockford, IL, USA) following the instructions of the manufacturer. Equal protein amounts were separated using 10% SDS–polyacrylamide gel electrophoresis and transferred onto a polyvinylidene difluoride membrane. The membranes were blocked with 5% nonfat milk at room temperature for 1 h and incubated overnight with primary antibodies (Santa Cruz Biotechnology, CA, USA). Horseradish peroxidase-conjugated anti-rabbit and anti-mouse antibodies (Santa Cruz Biotechnology) were used as secondary antibodies. Protein bands were detected using an enhanced chemiluminescence kit (Elpis Biotech Inc., Daejeon, Republic of Korea) and visualized via x-ray film exposure. Molecular weights were estimated using a prestained blue marker. Band intensities were quantified by densitometry using the ImageJ software (NIH, Bethesda, MD, USA). For each target protein, the integrated band intensity was normalized to that of glyceraldehyde-3-phosphate dehydrogenase from the same lane, and the normalized values were expressed relative to the TCPS control, which was set to 1.0. Quantification was performed on 4 independent blots per group, and the relative expression levels of the TGF-β/SMAD and Hippo-pathway components are presented as mean ± standard deviation.

### Statistical analysis

All quantitative data are presented as mean ± standard deviation. Unpaired Student *t* tests were used to analyze the cell adhesion, viability, and differentiation results. For comparisons involving 3 or more groups, one-way analysis of variance was conducted. *P* < 0.05 was considered statistically significant. Statistical analysis of the μ-CT data was performed using the Kruskal–Wallis test in the SPSS software.

## Results and Discussion

### Design and fabrication of the scaffolds

**Fig. 1.**
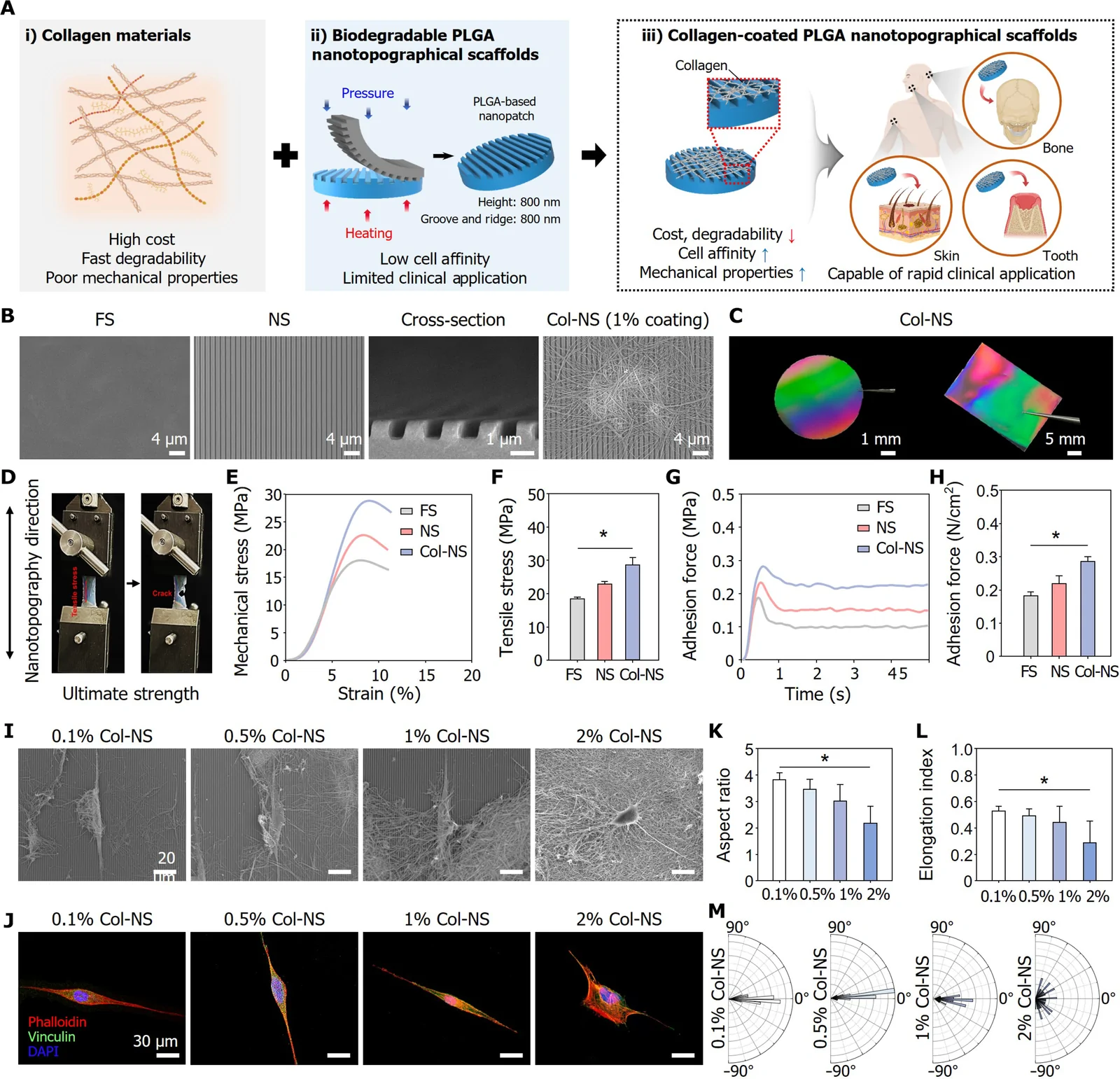
Design and characterization of Col-NS. (A) Inspired by the structural and biochemical complexity of the extracellular matrix (ECM), scaffolds were developed to integrate nanoscale topographical features with collagen-based biochemical signals to investigate their effect on hard- and soft-tissue regeneration. (B) Field-emission scanning electron microscopy (FE-SEM) images of FS, NS, and Col-NS. Scale bar: 4 μm. (C) Representative photograph of Col-NS. (D) Uniaxial tensile testing was performed to assess the mechanical properties of the scaffolds. (E and F) Stress–strain curves and tensile strength of the scaffolds. (G and H) Stress–strain curves and adhesion force measurements. (I) Representative FE-SEM images showing fibroblasts attached to the scaffolds. (J) Representative immunofluorescence images of fibroblasts cultured on the scaffolds, stained with 4′,6-diamidino-2-phenylindole (DAPI) (blue), vinculin (green), and F-actin (red). (K) Cell aspect ratio, (L) cell elongation index, and (M) angular distribution of cell orientation relative to the nanopattern direction for fibroblasts cultured on scaffolds coated with 0.1%, 0.5%, 1%, and 2% collagen, quantified from the low-magnification SEM and F-actin images in Fig. S1. Data are presented as mean ± SD. *P < 0.05, determined via one-way analysis of variance (ANOVA) followed by Tukey’s honestly significant difference (HSD) post hoc test.

Among the various biomaterial platforms used worldwide, biomedical membrane patches represent one of the most extensively applied systems in clinical tissue regeneration due to their ease of use, biocompatibility, and versatility across multiple tissue types. Despite their widespread clinical adoption, these conventional membrane patches have primarily played passive roles acting as physical barriers or wound coverings without intrinsic capacity to drive fundamental tissue regeneration. In this study, we introduce a bioactive biomedical membrane patch designed to extend beyond conventional passive barrier properties by integrating ECM-mimetic nanotopographical and biochemical cues. To achieve this, we designed a collagen-coated PLGA nanotopographical scaffold (Col-NS) by integrating biomimetic nanotopographical and biochemical cues that emulate the native ECM microenvironment. This rationally engineered scaffold provides structural support while modulating cellular and molecular processes associated with tissue repair and regenerative remodeling. Specifically, we developed Col-NS that integrates nanoscale ECM structural signals with collagen-based biochemical components to investigate their effects on human skin tissue and alveolar bone regeneration (Fig. 1A). The ECM is a dynamic, multifunctional network of proteins and molecules that surrounds cells and tissues, providing physical and chemical cues essential for regulating various biological functions [29,30]. Its biophysical and biochemical signals play crucial roles in modulating stem cell behaviors, including adhesion, proliferation, and differentiation [31]. The structural complexity of the ECM, comprising diverse geometries, shapes, and topologies formed through interactions among matrix components, spans nanoscale to microscale dimensions within cellular microenvironments [32]. Additionally, the collagen in ECM contributes substantially to structural support and cellular regulation. Specific ligands present in collagen molecules bind to cell surface receptors such as integrins to promote cell adhesion [33]. This adhesion is essential for guiding cell migration and plays a key role in tissue regeneration by inducing the necessary cellular movement [34]. Therefore, mimicking the hierarchical nanotopography of the ECM and incorporating biochemical components, such as collagen, are essential considerations when designing scaffolds for tissue regeneration.

As mentioned above, we investigated the effects of biochemically functionalized nanotopographical scaffolds on skin tissue regeneration based on the following rational design criteria: (a) The scaffolds should be composed of biodegradable and biocompatible materials that allow healing cells unrestricted access to the injury site during tissue regeneration. (b) The scaffolds should exhibit topographical cues and bioactive properties that support cell migration, proliferation, and differentiation, without disrupting normal cellular behavior. (c) The scaffolds should enhance tissue regeneration while minimizing toxicity, inflammation, and scarring. To satisfy these criteria, we fabricated aligned nanotopographical scaffolds using PLGA, a biocompatible, nontoxic, and biodegradable polymer.

The 800-nm ridge/groove dimension was selected based on our previous studies and established nanotopography literature, in which submicron aligned features (typically several hundred nanometers to ~1 μm) were shown to provide optimal contact guidance and cell alignment while remaining reproducibly fabricable by soft lithography [35–37]. Rather than replicating the exact diameter of individual collagen fibrils, this geometry approximates the anisotropic organization of bundled ECM fibrillar structures at the submicron-to-micron scale. Figure 1A illustrates the synergistic effects of the collagen coating and aligned nanotopographical scaffolds (NS). A PLGA solution was dispensed onto a nanotopographical PUA replica, covered with a flat PDMS block, and dried through solvent evaporation to fabricate the aligned nanotopographical scaffolds (NS). To assess nanotopographical effects, we developed scaffolds approximately 30 μm thick, featuring aligned nanotopography (ridge and groove: 800 nm; depth: 800 nm) or flat topographical scaffolds (FS) (Fig. 1A). Collagen solution was then applied to NS and evenly coated on the surface using spin coating. The surface of NS exhibited a highly aligned nanotopography with 800-nm-sized grooves and ridges, providing the well-organized, anisotropic contact-guidance cues characteristic of the native ECM microenvironment (Fig. 1B). This dimension falls within the nano-to-microscale range over which ECM fibrillar structures are organized and was chosen based on prior reports of effective cell alignment and tissue regeneration on submicron aligned topographies. The collagen coating (1% w/v in distilled water) resulted in the uniform deposition of collagen fibers onto the nanotopographical surface. The fabricated scaffolds were freestanding and flexible, capable of conforming to various structures, including the irregular surfaces of biological tissues or organs, making them suitable for transplantation use (Fig. 1C).

The stress–strain behavior and surface adhesion forces of the scaffolds with flat, nanotopographical, and collagen-coated nanotopographical surfaces were analyzed (Fig. 1D to H). During tensile testing, mechanical loading was applied along the orientation of the nanotopography in Col-NS (Fig. 1D). When the load was aligned with the direction of the nanotopography, NS exhibited greater tensile stress (23.06 MPa) than that of FS (17.65 MPa) (Fig. 1E and F). Furthermore, Col-NS showed the highest tensile stress (29.86 MPa) compared to that of the uncoated NS. The nanoscale surface roughness of NS, combined with the hydrophilic properties induced via collagen coating, enhanced adhesion through a mechanism similar to van der Waals forces (Fig. 1G and H). Overall, the incorporation of aligned nanotopographical structures and collagen coating markedly improve the mechanical properties of NS, demonstrating superior performance compared to that of FS.

To determine the optimal collagen concentration that preserves the highly aligned nanotopography of the coated scaffold, collagen solutions at 0.1%, 0.5%, 1%, and 2% were applied to the nanotopographical surfaces (Fig. 1I). At a 1% collagen concentration, the nanotopography was partially covered, whereas at 2%, the surface was completely masked by collagen fibers. Fibroblasts were cultured on Col-NS for 24 h, and the samples were visualized using scanning electron microscopy (SEM) and immunofluorescence staining (Fig. 1I and J). Cells cultured on surfaces coated with 0.1%, 0.5%, and 1% collagen responded to the nanotopography between the collagen fibers, aligning unidirectionally along the nanotopography. In contrast, cells on surfaces coated with 2% collagen adhered randomly across the uniformly covered surface, independent of the nanotopographical structure. This concentration-dependent behavior reveals a functional trade-off between the 2 design cues. Although collagen supplies biochemical adhesion signals, coating densities beyond approximately 1% progressively bury the 800-nm relief, and at 2%, the anisotropic contact-guidance effect was markedly attenuated, causing cells to revert to nondirectional adhesion (Fig. S1A and B). The biological response is therefore governed by both elements in a concentration-dependent manner. Topographical guidance predominates only while the surface relief remains exposed, whereas excess collagen shifts the response toward coating-driven, nonoriented adhesion. These findings identify 1% collagen as the optimal concentration because it preserves exposed topographical cues for contact guidance while providing sufficient collagen-derived biochemical signaling.

To quantitatively corroborate these concentration-dependent responses at the population level, fibroblasts cultured on scaffolds coated with 0.1%, 0.5%, 1%, and 2% collagen were imaged at low magnification by SEM and F-actin (phalloidin) staining across the full concentration series, allowing the morphology and alignment of multiple cells to be assessed simultaneously within each field (Fig. S1A and B). Their morphology and orientation were then quantified as cell aspect ratio, elongation index, and angular distribution relative to the nanopattern direction (Fig. 1K to M). Both the SEM and F-actin images showed that the majority of cells on the 0.1%, 0.5%, and 1% collagen surfaces adopted an elongated, spindle-like morphology and extended collectively parallel to the underlying nanopattern direction, whereas cells on the 2% collagen surface spread in a flattened, randomly oriented manner over the collagen-masked surface, losing directional guidance. Consistent with these observations, the cell aspect ratio decreased progressively with increasing collagen concentration, from 3.8 at 0.1% to 3.4, 3.0, and 2.2 at 0.5%, 1%, and 2%, respectively (Fig. 1K). The cell elongation index followed the same trend, declining from 0.53 at 0.1% to 0.49, 0.44, and 0.30 at 0.5%, 1%, and 2%, respectively (Fig. 1L). These results indicate that fibroblasts maintained a highly polarized, elongated shape while the nanotopography remained accessible at lower collagen densities but shifted toward a rounder, less polarized morphology once the surface relief was masked at 2% collagen. The orientation analysis reinforced this trade-off (Fig. 1M). On surfaces coated with 0.1%, 0.5%, and 1% collagen, the major axes of the cells were tightly distributed around 0°, reflecting the strong unidirectional alignment of the cell population along the nanopattern direction. In contrast, cells on the 2% collagen-coated surface exhibited a broad, nearly isotropic angular distribution, consistent with the loss of contact-guidance cues once the nanotopography was buried beneath the collagen layer. Collectively, these quantitative morphological and orientational data confirm that fibroblast elongation and alignment are dictated by the accessibility of the underlying nanotopography and reinforce that 1% collagen represents the optimal coating density that preserves topographical guidance while providing sufficient collagen-derived biochemical signaling. Vinculin immunostaining corroborated these findings at the focal adhesion level (Fig. S2A). Vinculin orientation was tightly aligned around 0° at 0.1%, 0.5%, and 1% collagen but broadly dispersed at 2% (Fig. S2B), and the cell aspect ratio remained high across 0.1% to 1% before dropping sharply at 2% (Fig. S2C), confirming that the elongated, contact-guided morphology persisted only while the nanotopography was exposed. The focal adhesion area increased modestly with collagen concentration, being the largest at 1% and 2% (Fig. S2D), consistent with enhanced integrin-mediated adhesion from the collagen coating. These data reinforce 1% collagen as the optimal balance between topographical guidance and adhesive signaling.

### Clinical case study: Skin tissue regeneration

**Fig. 2.**
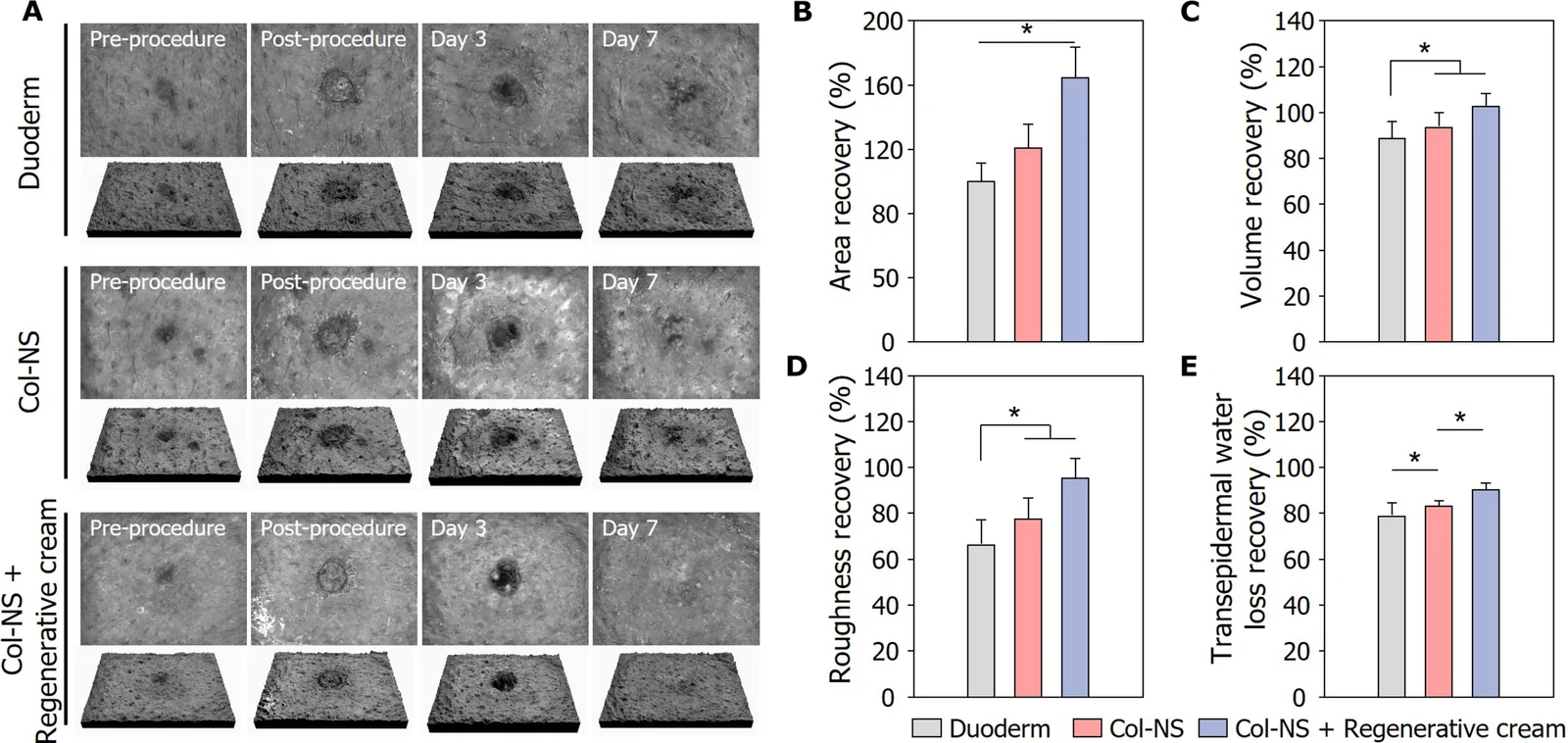
Clinical evaluation of human skin regeneration using Col-NS. (A) Representative clinical photographs showing skin regeneration after laser treatment at 4 time points: pre-procedure, post-procedure, and days 3 and 7. Images show comparative outcomes across 3 groups: Duoderm, Col-NS, and Col-NS combined with a regenerative cream. (B to E) Quantitative analyses of area, volume, surface roughness, and transepidermal water loss (TEWL) recoveries. Data are presented as mean ± SD. *P < 0.05, determined via one-way analysis of variance (ANOVA) followed by Tukey’s HSD post hoc test.

To evaluate the clinical efficacy of Col-NS in enhancing skin regeneration after laser-induced injury, we analyzed changes in 4 major parameters: wound area, depression volume, surface roughness, and TEWL. Assessments were conducted at 4 time points: baseline (pretreatment), immediately after laser treatment, and on days 3 and 7 following product application. Three groups were compared: the Duoderm patch (control), Col-NS, and Col-NS combined with regenerative cream. All parameters showed statistically significant improvement over time (*P* < 0.05), and the scaffold groups, particularly those combined with regenerative cream, generally exhibited greater therapeutic effects than the control (Fig. 2A).

Analysis of wound area using the Antera 3D imaging system revealed substantial reductions across all 3 groups (Fig. 2B). In the Duoderm patch group, the wound area decreased from 495.6 arbitrary units (A.U.) postlaser to 367.6 A.U. on day 3 and 209.2 A.U. on day 7, corresponding to improvement rates of 39.72% and 100.26%, respectively. In the Col-NS group, the wound area decreased from 491.5 to 376.7 and 210.9 A.U. on days 3 and 7, with improvement rates of 43.41% and 122.19%, respectively. Col-NS combined with regenerative cream showed the most pronounced effect, with the wound area decreasing from 456.4 to 363.0 and 170.4 A.U., reflecting improvement rates of 38.81% and 163.02%, respectively. Depression volume analysis also revealed substantial reductions in all groups, indicating enhanced dermal tissue recovery (Fig. 2C). In the Duoderm patch group, the depression volume declined from 0.287 to 0.091 and 0.060 mm^3^ on days 3 and 7, yielding improvement rates of 76.13% and 91.67%, respectively. In the Col-NS group, the volume decreased from 0.263 to 0.069 and 0.044 mm^3^, with improvement rates of 78.96% and 93.02%, respectively. In the group with scaffolds and regenerative cream, the volume was reduced from 0.283 to 0.083 and 0.045 mm^3^, resulting in improvement rates of 77.10% and 103.53%, respectively. Skin surface roughness (R3) also demonstrated statistically significant decreases in all 3 groups (Fig. 2D). The Duoderm patch group exhibited a reduction from 67.253 to 54.548 A.U. on day 3 and 49.594 A.U. on day 7, with improvement rates of 43.09% and 66.38%, respectively. The Col-NS group showed a reduction in surface roughness from 71.769 to 58.096 A.U. on day 3 and 48.146 A.U. on day 7, indicating improvement rates of 41.61% and 79.10%, respectively. Col-NS combined with regenerative cream demonstrated the greatest improvement, with roughness decreasing from 69.465 to 56.183 and 46.034 A.U., resulting in improvement rates of 44.85% and 95.52%, respectively. TEWL values were also substantially reduced across all groups, indicating improved skin barrier recovery (Fig. 2E). In the Duoderm patch group, TEWL reduced from 69.540 to 43.503 g/m^2^ h on day 3 and 30.659 g/m^2^ h on day 7, with improvement rates of 53.33% and 79.84%, respectively. In the Col-NS group, values decreased from 70.520 to 50.472 and 29.583 g/m^2^ h, with improvement rates of 40.29% and 82.03%, respectively. The Col-NS combined with regenerative cream group achieved the highest reduction, from 69.804 to 49.114 and 25.637 g/m^2^ h, resulting in improvement rates of 42.78% and 91.98%, respectively. No adverse dermatologic reactions were observed in any participants throughout the evaluation period, confirming the safety of all treatment modalities.

Taken together, these findings indicate that Col-NS effectively promotes skin regeneration following laser-induced injury. Improvements were consistently observed across all parameters, including wound contraction, tissue volume restoration, surface smoothness, and skin barrier function. The combination of Col-NS and regenerative cream yielded the most pronounced therapeutic outcomes, suggesting a synergistic effect. These findings support the clinical potential of Col-NS as a next-generation platform for advanced skin repair.

### Clinical case study: Alveolar bone regeneration

#### Description of cases

All surgical procedures were performed by a single experienced periodontist (W.-P.L.). Before surgery, patients were rinsed with 0.12% chlorhexidine solution for 1 min. Local anesthesia was administered using 2% lidocaine with 1:100,000 epinephrine. In GBR cases, a full-thickness flap was elevated following a crestal incision with vertical releasing incisions using a #15 blade to ensure adequate bone exposure. In contrast, ARP was performed employing a flapless or minimally invasive technique. In these cases, the extraction socket was filled with particulate bone graft material and covered with Col-NS, applied directly without primary closure. Primary closure was obtained in GBR procedures via flap advancement, whereas in ARP cases, the membrane was intentionally left exposed.

Postoperative care included a 7-d course of oral antibiotics (Augmentin 625 mg; Ilsung Pharm. Co., Seoul, Korea) and analgesics (aceclofenac 100 mg; Dona-A ST, Seoul, Korea). Patients were instructed to rinse twice daily with 0.12% chlorhexidine for 2 weeks, and sutures were removed at the 2-week follow-up.

#### Clinical and radiological findings

In total, 17 implants were placed in 10 patients who underwent either ARP or GBR using Col-NS. All cases exhibited uneventful healing, with no signs of infection, membrane exposure in GBR, or dehiscence. Implants were restored after a healing period of 3 to 5 months, depending on the surgical indication. During functional loading, implant sites were monitored using periapical and panoramic radiographs.

**Fig. 3.**
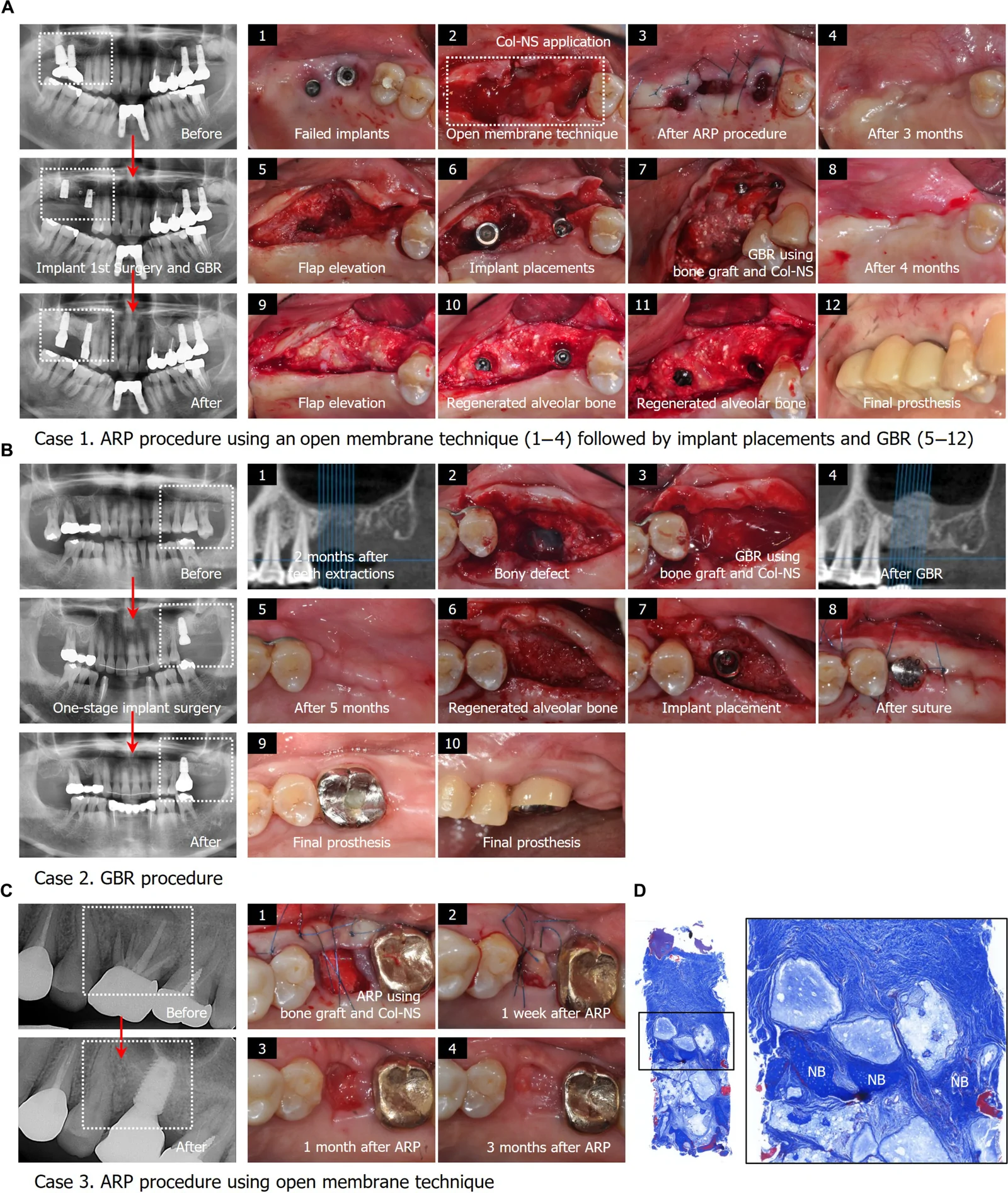
Clinical evaluation of human alveolar bone regeneration using Col-NS. (A) Clinical cases demonstrating alveolar bone regeneration under severe conditions: case 1, implant placement following alveolar ridge preservation (ARP) using an open membrane technique and the guided bone regeneration (GBR) procedure; (B) case 2, GBR procedure; and (C) case 3, ARP with an open membrane technique. (D) Representative Masson’s trichrome (MT)-stained histological images.

Based on the International Congress of Oral Implantologists Pisa Consensus criteria, all implants were classified as successful, showing no peri-implant radiolucency or marginal bone loss. Clinical evaluation revealed consistently healthy, keratinized mucosa surrounding each implant site. Graft volume remained stable, with no clinically substantial reduction in ridge height or width. These findings support the predictable regenerative performance of Col-NS in open and closed surgical contexts (Fig. 3A to C).

#### Histological findings of the clinical study

Histological evaluation of biopsied specimens from the ARP-treated and GBR-treated sites using Col-NS revealed substantial new bone formation in close contact with residual graft particles. The newly formed bone exhibited mature lamellar structure with evidence of vascularization and showed no signs of inflammatory cell infiltration or foreign body reaction, indicating the excellent biocompatibility of Col-NS.

In a representative case, a 67-year-old female patient underwent ARP at site #26 using an octacalcium phosphate-based synthetic bone graft in combination with Col-NS via the open membrane technique. At 3 months, a core biopsy was obtained during implant placement for histological analysis. Histomorphometric evaluation revealed that new bone formation accounted for 21.3% of the total tissue volume, while residual graft material occupied 32.2%. MT staining revealed newly formed bone beneath the overlying soft tissue, closely integrated with the surrounding synthetic graft particles. Multiple layers of osteoid were observed lining the periphery of the graft particles, indicating active osteogenesis. No inflammatory infiltrates or foreign body reactions were detected, further supporting the excellent tissue biocompatibility of Col-NS (Fig. 3D).

These histological findings validate that Col-NS, even under nonsubmerged, open healing conditions, can effectively support early-phase bone regeneration and promote the formation of osteoid and vascularized lamellar bone without eliciting adverse tissue responses. The implant achieved successful osseointegration and prosthetic loading without complications.

Explantation of the biodegradable polymeric, nanotopographical scaffold following the GBR period was performed to enable ex vivo assessment of device integrity and overlying tissue morphology (Fig. S3). At 3 months postplacement, the retrieved Col-NS maintained its macroscopic mechanical integrity and gross geometry, with no observable evidence of bulk degradation or structural collapse (Fig. S3A). High-resolution SEM confirmed the complete preservation of the engineered nanotopographical features (Fig. S3B). The tissues adherent to the scaffold surface exhibited contact guidance, characterized by the anisotropic organization of both cellular and ECM constituents (Fig. S3C). Specifically, the ECM displayed parallel alignment and increased packing density along the axis of the nanotopography orientation. These findings indicate that the nanotopography sustained structural integrity in vivo and furnished persistent directional cues. Consequently, these cues promoted denser, more uniformly oriented ECM formation, consistent with topography-mediated enhancement of osteoconductive matrix assembly during alveolar bone regeneration.

### Skin regeneration effect of the scaffolds in the normal animal model

**Fig. 4.**
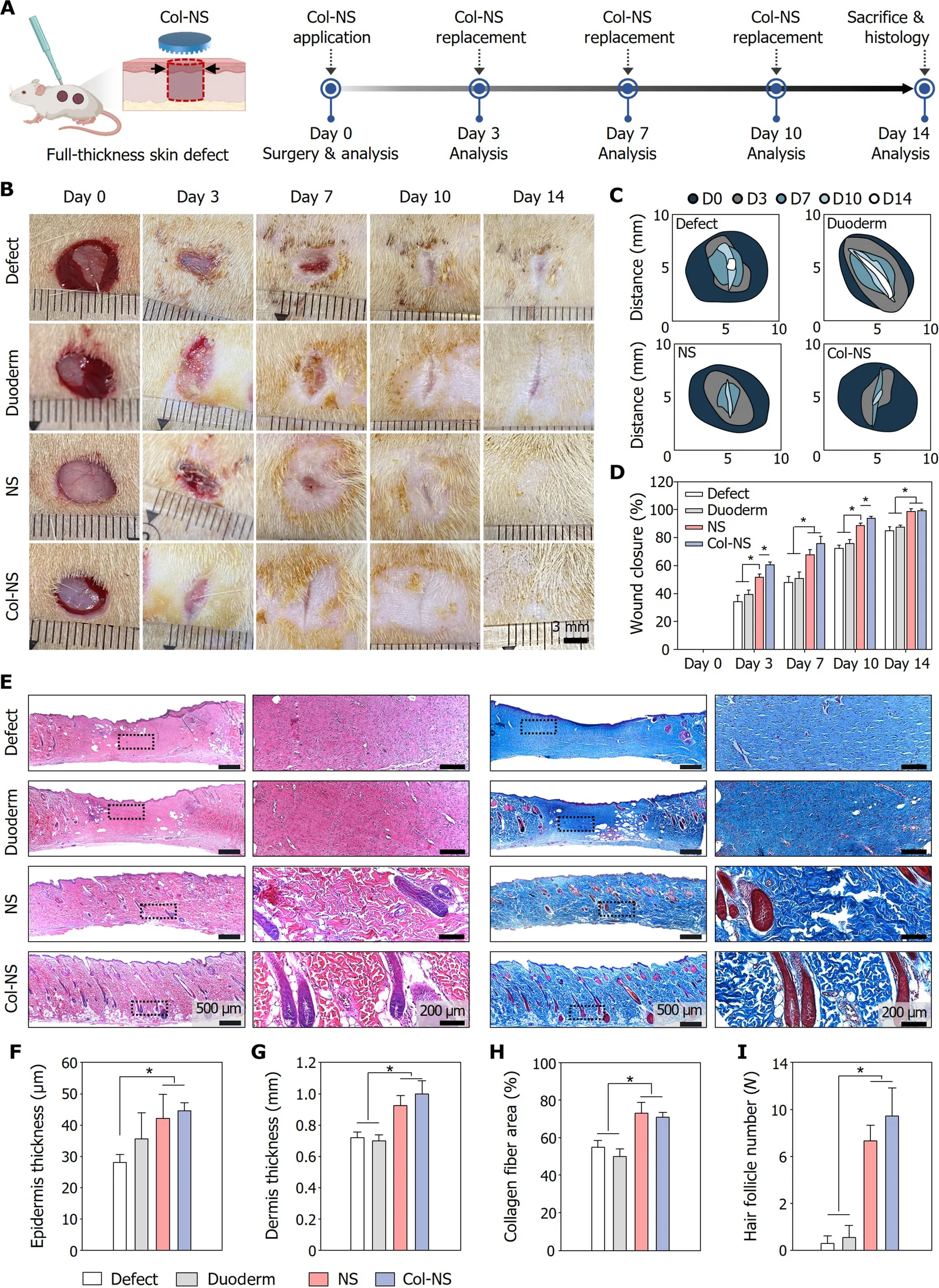
In vivo skin regeneration study using a full-thickness skin defect model in rats. (A) Schematic timeline of the experimental procedure. (B) Macroscopic images of wound healing progression on days 0, 3, 7, 10, and 14 in 4 groups: defect, Duoderm, NS, and Col-NS. (C and D) Quantitative analysis of wound-closure percentages for the different scaffold groups. (E) Representative histological images of hematoxylin and eosin (H&E) and Masson’s trichrome (MT) staining at the wound site. Scale bar: 200 μm. (F to I) Quantitative histological analyses of epidermal thickness, dermal thickness, collagen fiber deposition, and hair follicle count. Data are presented as mean ± SD. *P < 0.05, determined via one-way analysis of variance (ANOVA) followed by Tukey’s HSD post hoc test.

The effects of Col-NS on skin regeneration were investigated using a full-thickness skin defect rat model. Four experimental groups were established: untreated defects, wounds treated with Duoderm (a commercially available skin dressing), NS, and Col-NS (Fig. 4). Wound images were captured on days 0, 3, 7, 10, and 14 to evaluate wound-closure rates (Fig. 4A). The percentage of wound closure varied among groups and across time points (Fig. 4B). On day 7, the closure rates were 47.94%, 50.55%, 67.56%, and 75.55% for the untreated, Duoderm, NS, and Col-NS groups, respectively (Fig. 4C and D). By 14 d, these rates increased to 84.81%, 87.18%, 98.33%, and 99.12%, respectively (Fig. 4C and D). Wounds treated with Col-NS showed substantially faster healing than the other groups. Notably, the uncoated NS group already achieved markedly higher closure than Duoderm at day 7 (67.56% vs. 50.55%), and the collagen coating provided a further increment (Col-NS, 75.55%), indicating that the aligned nanotopography accounts for the major share of the wound-closure benefit in vivo, with the collagen coating offering an additive contribution.

The wound healing effects of Col-NS were further assessed through histological analysis using H&E and MT staining (Fig. 4E). Quantitative measurements were performed for epidermal thickness, dermal thickness, collagen fiber deposition, and hair follicle count (Fig. 4F to I). Skin tissues regenerated with Col-NS showed thick, well-structured layers, resembling native skin. In contrast, tissues from the untreated, Duoderm, and NS groups exhibited thinner epidermal and dermal layers with minimal collagen fiber formation. Newly formed hair follicles were observed in the dermal layers of the Col-NS group, suggesting enhanced tissue regeneration compared to that of the other groups. These follicles are presumed to originate from epithelial cells as healing progresses. Collectively, these findings indicate that Col-NS provides the most effective skin repair and tissue reconstruction by mimicking the structural and functional characteristics of native skin.

### Skin regeneration effect of the scaffolds in diabetic animal models

**Fig. 5.**
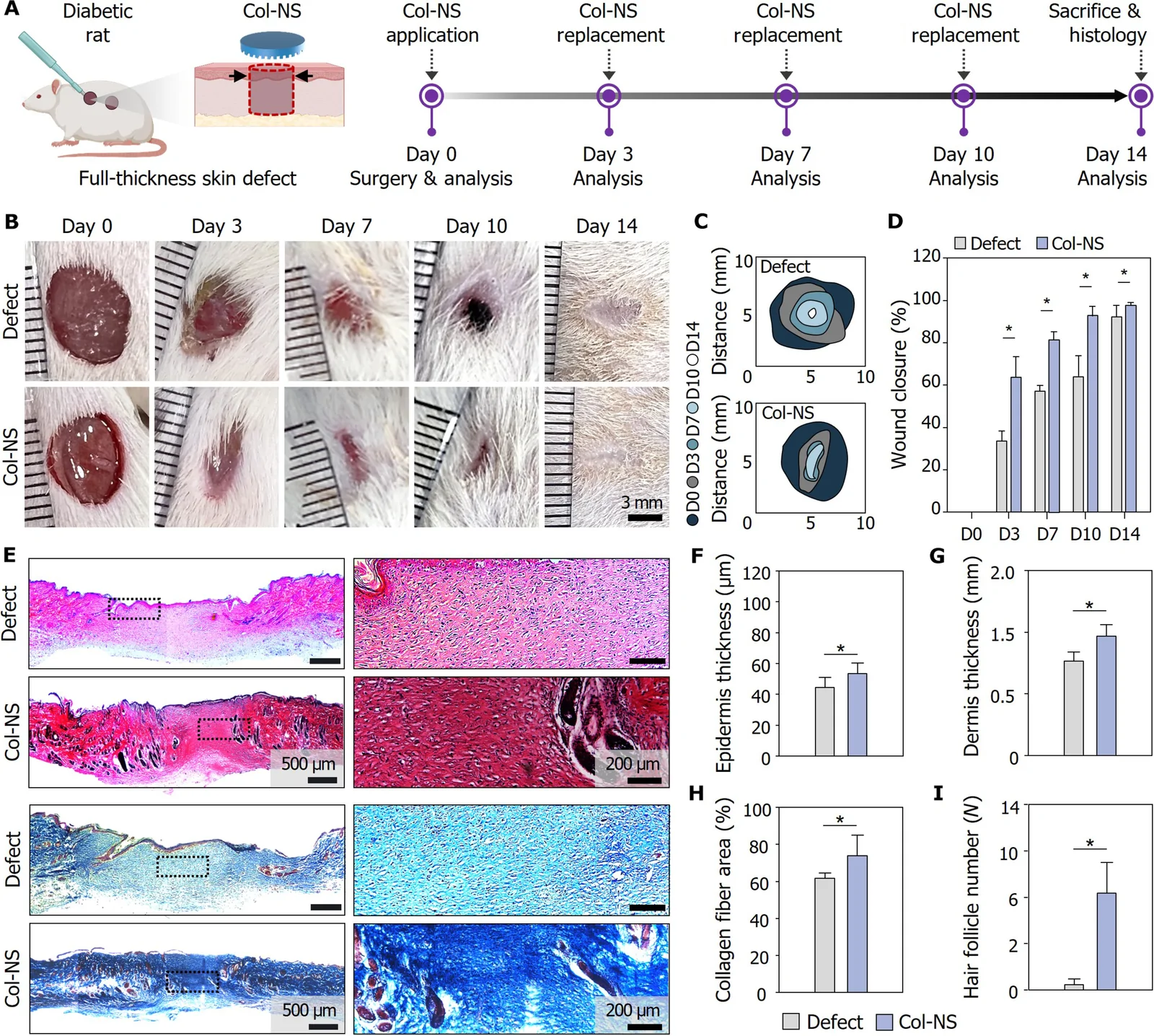
In vivo skin regeneration study using a full-thickness skin defect model in diabetic rats. (A) Schematic timeline of the experimental procedure. (B) Macroscopic images showing wound healing progression on days 0, 3, 7, 10, and 14. (C and D) Quantitative analysis of wound-closure percentages across the experimental groups. (E) Representative histological images of hematoxylin and eosin (H&E) and Masson’s trichrome (MT) staining at the wound site. Scale bar: 200 μm. (F to I) Quantitative histological assessments of epidermal thickness, dermal thickness, collagen fiber formation, and hair follicle count. Data are presented as mean ± SD. *P < 0.05, determined through one-way analysis of variance (ANOVA) followed by Tukey’s HSD post hoc test.

The efficacy of Col-NS on skin regeneration was assessed using a full-thickness skin defect diabetic rat model (Fig. 5). Two experimental groups were established: an untreated defect group and a Col-NS group (Fig. 5A). Wound images were captured on days 0, 3, 7, 10, and 14 to evaluate wound-closure rates (Fig. 5B). The extent of wound closure varied between groups at each time point. On day 7, the closure rates were 57.42% and 80.26% for the untreated and Col-NS groups, respectively (Fig. 5C and D). By day 14, these values increased to 92.37% and 99.45%, respectively (Fig. 5C and D). The wounds in the group treated with Col-NS exhibited substantially faster healing.

Histological analyses were performed to evaluate wound healing using H&E and MT staining (Fig. 5E). Quantitative measurements included epidermal thickness, dermal thickness, collagen fiber deposition, and hair follicle formation (Fig. 5F to I). Skin tissues regenerated with Col-NS exhibited thick, well-organized layers resembling native skin. Conversely, tissues in the untreated group showed thinner epidermal and dermal layers with limited collagen fiber formation. Newly formed hair follicles were observed in the dermal layers treated with Col-NS, indicating improved tissue regeneration compared to that of the untreated group. These follicles are hypothesized to originate from epithelial cells as healing progresses. Collectively, Col-NS demonstrated the most effective skin recovery and tissue reconstruction in the diabetic model, closely reflecting the properties of native skin. These findings underscore the potential of Col-NS as a promising therapeutic strategy for enhancing wound healing in diabetic conditions.

### Bone regeneration effect of the scaffolds in animal models

**Fig. 6.**
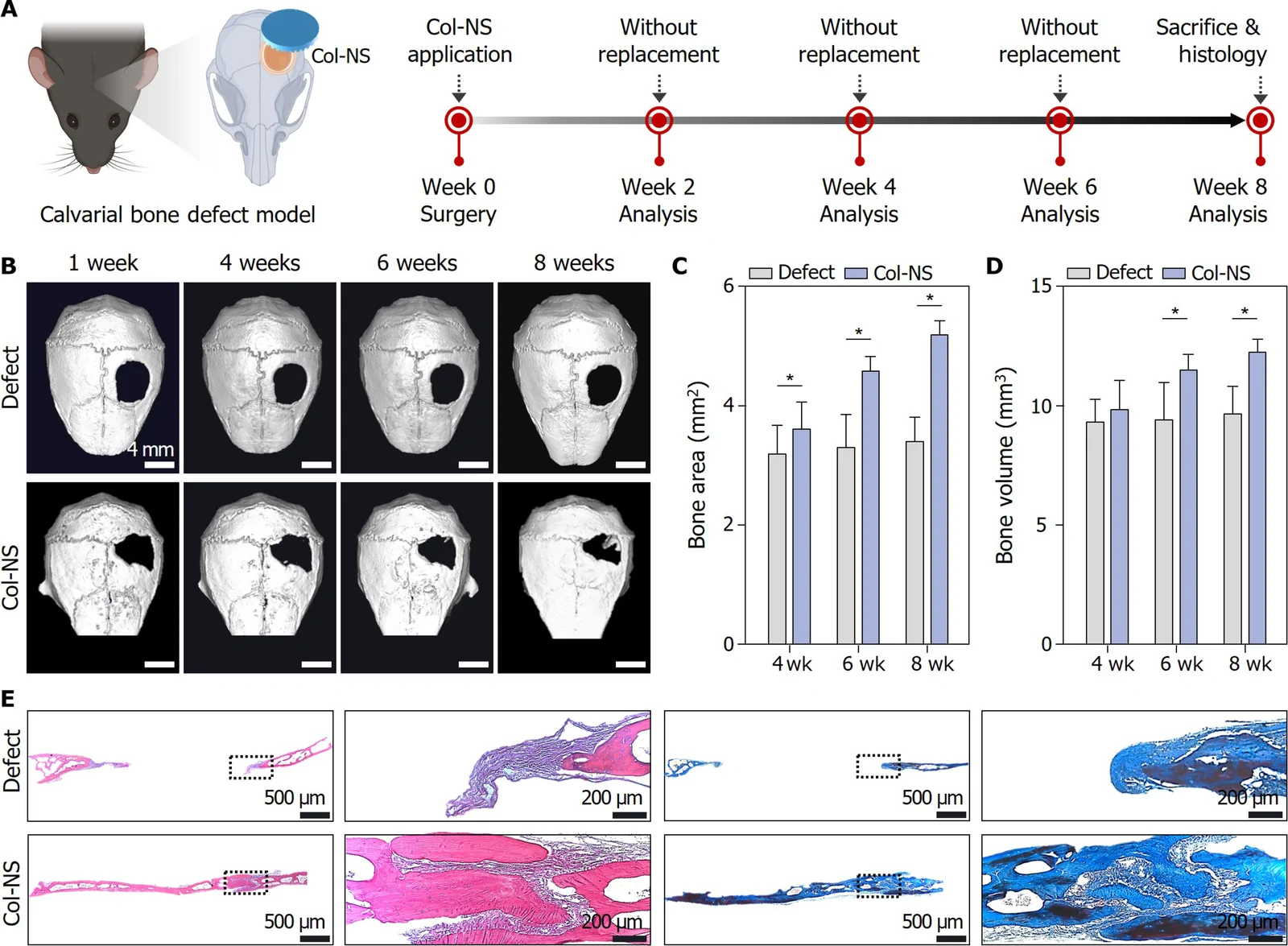
In vivo bone regeneration using calvarial bone defect models. (A) Schematic timeline of the experimental procedure. (B) Representative micro-computed tomography (μ-CT) images at weeks 1, 4, 6, and 8. (C) Quantitative bone area analysis of μ-CT images after 4, 6, and 8 weeks. (D) Quantitative bone volume analysis of μ-CT images after 4, 6, and 8 weeks. (E) Representative histological micrographs stained with hematoxylin and eosin (H&E) and Masson’s trichrome (MT). Data are presented as mean ± SD. *P < 0.05, **P < 0.01, and ***P < 0.001, determined through one-way analysis of variance (ANOVA) followed by Tukey’s HSD post hoc test.

All mice used in the in vivo experiments survived until the designated euthanasia time point, with no adverse effects observed throughout the study. Col-NS (7 mm in diameter) was implanted into surgically induced calvarial bone defects. No signs of infection or inflammation were detected during the postoperative period. In vivo outcomes confirmed the effectiveness of Col-NS in promoting bone regeneration (Fig. 6A). The scaffolds remained structurally intact without deformation for up to 8 weeks postimplantation. To quantitatively evaluate the effects of Col-NS on bone formation, new bone formation was analyzed using μ-CT and 3D reconstruction with the MIMICS 14.0 software. The 3D images (Fig. 6B) show that bone formation in the Col-NS group occurred at the defect margins and progressed centripetally, following the direction of the nanotopography. No complete bone formation was observed in the untreated group from week 1 to week 8, whereas the Col-NS group showed substantially enhanced bone regeneration throughout the implantation period. At 8 weeks, the bone area measured 3.11 and 5.15 mm^2^ in the untreated and Col-NS groups, respectively (Fig. 6C). Bone volume was also greater in the scaffold group (12.46 mm^3^) than in the untreated group (9.21 mm^3^) (Fig. 6D). To further assess the bone regeneration efficacy, H&E staining was performed at week 8 (Fig. 6E). Histological analysis revealed more extensive bone tissue formation and denser tissue structures in the Col-NS group than in the defect group. These findings highlight the critical role of collagen coating combined with nanoscale topographical cues in promoting bone regeneration.

### Effect of the scaffolds on cellular behaviors and responses

**Fig. 7.**
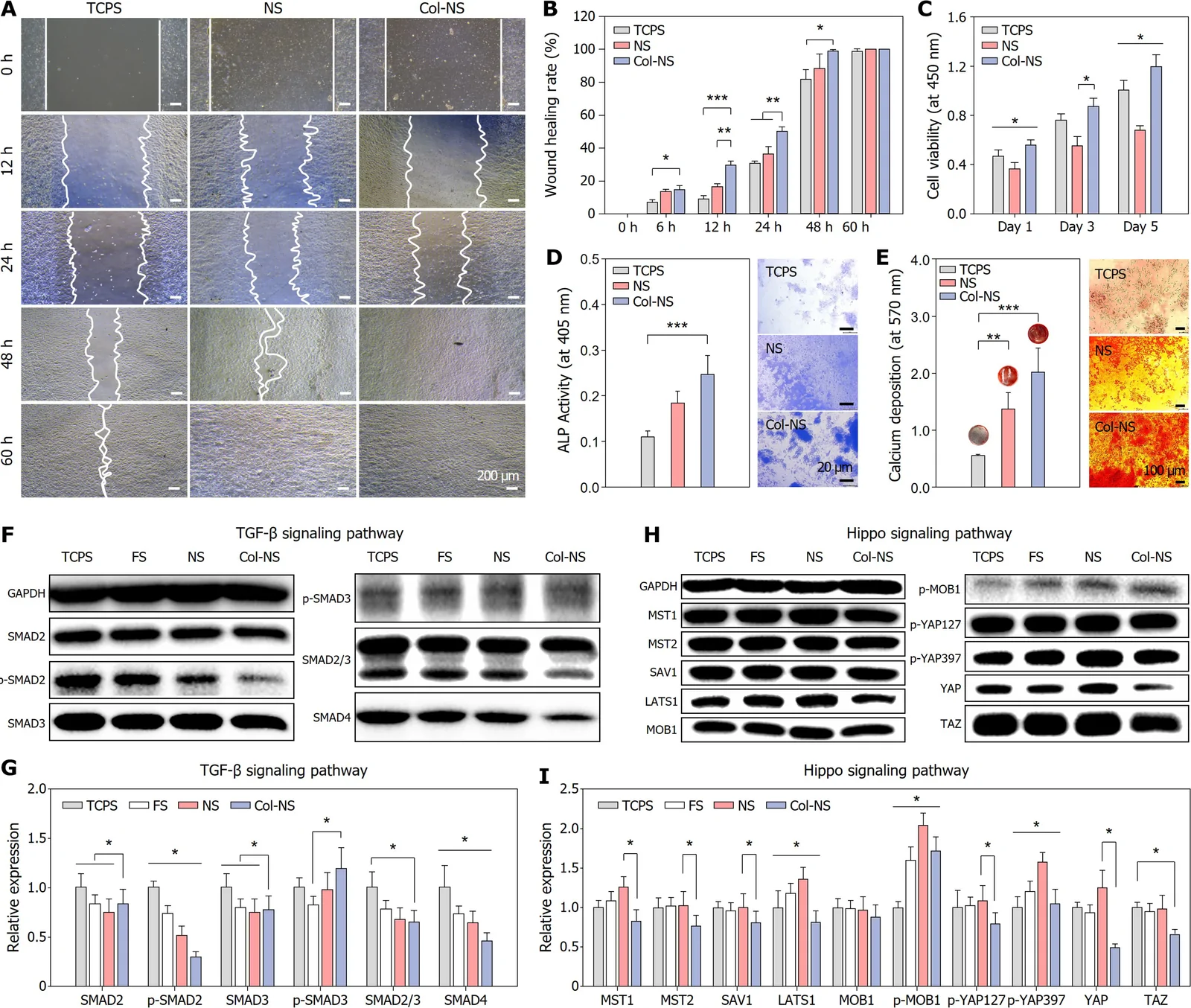
Mechanistic study of the effects of Col-NS on cellular behaviors and responses. (A) Representative images of the NIH 3T3 fibroblast migration assay at 0, 12, 24, 48, and 60 h. (B) Quantitative analysis of the migration rate of NIH 3T3 fibroblasts. (C) Viability of NIH 3T3 fibroblasts assessed using the WST-1 assay. (D) Alkaline phosphatase (ALP) activity as an indicator of early osteogenic differentiation in MG-63 cells. (E) Alizarin Red S staining for the assessment of calcium deposition and mineralization in MG-63 cells. (F) Representative Western blots of the transforming growth factor-β (TGF-β)/SMAD signaling pathway in NIH 3T3 fibroblasts. (G) Quantitative analysis of the TGF-β/SMAD signaling proteins. (H) Representative Western blots of the Hippo–YAP/TAZ signaling pathway, including yes-associated protein (YAP) and transcriptional coactivator with PDZ-binding motif (TAZ). (I) Quantitative analysis of the Hippo–YAP/TAZ signaling proteins. All band intensities were normalized to glyceraldehyde-3-phosphate dehydrogenase (GAPDH) and expressed relative to the tissue culture polystyrene (TCPS) control. Data are presented as mean ± SD. *P < 0.05, **P < 0.01, and ***P < 0.001, determined via one-way analysis of variance (ANOVA) followed by Tukey’s HSD post hoc test.

To assess the effects of aligned nanostructures and collagen coating on in vitro cell migration, a thin PDMS slab was placed on the scaffold substrate, and NIH 3T3 fibroblasts were cultured on the substrate for 24 h (Fig. 7A). The PDMS slab was then removed to create a cell-free zone, and cell migration into this area was monitored using optical microscopy. Col-NS demonstrated substantially faster cell migration than the other groups, completely covering the cell-free area within 48 h (Fig. 7B). After 48 h, the closure rates for TCPS, uncoated NS, and Col-NS were 81.54%, 85.12%, and 100%, respectively. Whereas nanotopography alone (NS vs. TCPS) produced only a modest increase (~3.6%), the collagen coating contributed the largest incremental gain (~14.9%; NS vs. Col-NS), indicating that migration in this assay is driven predominantly by the biochemical coating rather than by the structural cue.

Biocompatibility is a critical factor for the biomedical application of Col-NS. To investigate its biological activity, NIH 3T3 fibroblasts were used to assess cell adhesion and proliferation (Fig. 7C). WST-1 assays were conducted on days 1, 3, and 5. Cell adhesion and proliferation were the highest in Col-NS. This enhanced performance can be attributed to the collagen coating, which creates a biomimetic, cell-supportive microenvironment resembling the ECM, thereby promoting cell adhesion and growth. These findings highlight the potential of Col-NS as an advanced platform for tissue engineering applications, emphasizing its ability to support rapid wound closure and enhance cell proliferation.

To evaluate ALP activity as a marker of early osteogenic differentiation, MG-63 cells were cultured on 2 scaffold types in osteogenic induction medium for 7 d (Fig. 7D). ALP activity revealed a stepwise increase in osteogenic activity from TCPS to uncoated NS and to Col-NS, with the collagen coating providing the largest increment over nanotopography alone, indicating that osteogenic differentiation depends primarily on the collagen-derived biochemical cue, while the aligned topography provides a supporting structural contribution. Furthermore, to assess osteogenic mineralization, MG-63 cells were cultured under the same conditions for 14 d. Alizarin Red S staining (Fig. 7E) revealed increased calcium deposition on Col-NS compared to that of the other groups. ALP staining (indicative of early osteogenic differentiation) and Alizarin Red S staining (representing late-stage osteogenic maturation) consistently revealed enhanced osteogenic potential in Col-NS.

TGF-β and Hippo signaling pathways are functionally interconnected regulatory networks that coordinate cell proliferation, differentiation, migration, ECM remodeling, inflammation, and tissue-level mechanotransduction [38]. These pathways are particularly important during wound healing because their temporal and spatial regulation determines whether tissue repair proceeds toward regenerative remodeling or fibrotic scar formation. In the early phase of wound healing, TGF-β signaling is activated through SMAD-dependent cascades, in which receptor-mediated phosphorylation of SMAD2/3 promotes their association with SMAD4 and subsequent nuclear translocation. This transcriptional complex induces the expression of profibrotic genes associated with fibroblast activation, myofibroblast differentiation, collagen deposition, ECM accumulation, and inflammatory amplification [39]. Therefore, excessive or prolonged activation of the TGF-β/SMAD axis is widely recognized as a key molecular driver of fibrosis and scar formation.

Consistent with this concept, Western blot analysis demonstrated a graded attenuation of TGF-β/SMAD signaling across the groups (Fig. 7F). The reduction in SMAD2/3, p-SMAD2, and SMAD4 was already evident in uncoated NS relative to flat and TCPS controls and was most pronounced in Col-NS, indicating that the aligned nanotopography initiates suppression of profibrotic SMAD signaling through contact-guided cytoskeletal organization, while the collagen coating further amplifies this effect. Because p-SMAD2 reflects the activated state of canonical TGF-β signaling and SMAD4 functions as a central co-SMAD required for downstream transcriptional responses, their down-regulation indicates that Col-NS suppresses SMAD-mediated profibrotic signaling. This reduction may limit fibroblast overactivation, excessive ECM synthesis, and collagen-rich scar matrix deposition. Notably, the suppressive effect was more pronounced in Col-NS than in uncoated NS, suggesting that the combination of ECM-mimetic nanotopographical guidance and collagen biochemical cues provides a more favorable microenvironment for regenerative remodeling rather than fibrotic repair. Quantitative analysis supported these observations (Fig. 7G). Relative to the TCPS control, Col-NS substantially reduced the levels of p-SMAD2 (to ~0.30-fold), SMAD4 (~0.46), and SMAD2/3 (~0.65), along with modest decreases in total SMAD2 (~0.85) and SMAD3 (~0.79), following a graded trend across TCPS > FS ≈ NS > Col-NS for most components. The strongest suppression occurred in the activated effector p-SMAD2 and the central co-SMAD SMAD4, consistent with attenuation of canonical, profibrotic TGF-β/SMAD signaling. In contrast, p-SMAD3 was modestly increased in Col-NS (~1.2-fold), indicating that the suppression was selective for the p-SMAD2–SMAD4 arm rather than a uniform reduction of all SMAD proteins.

In parallel, the Hippo signaling pathway regulates tissue growth, cell density sensing, cytoskeletal tension, migration, and mechanotransduction through its downstream effectors yes-associated protein (YAP) and transcriptional coactivator with PDZ-binding motif (TAZ). Dysregulated YAP/TAZ activity has been strongly associated with fibroblast activation, myofibroblast transition, matrix stiffening, ECM overproduction, and persistent scar formation [40]. Under regenerative conditions, balanced Hippo–YAP/TAZ signaling supports controlled cell proliferation and migration, whereas excessive YAP/TAZ activation promotes fibrotic tissue remodeling. Therefore, regulation of this pathway is essential for achieving structurally organized and scar-minimized wound repair.

Western blot analysis showed that Col-NS altered the abundance of multiple Hippo-pathway-associated proteins (Fig. 7H). Most notably, the terminal mechanotransductive effectors YAP and TAZ were markedly reduced in Col-NS relative to the other groups. Several upstream cascade components, including mammalian STE20-like kinase 1 (MST1), mammalian STE20-like kinase 2 (MST2), Salvador homolog 1 (SAV1), and large tumor suppressor 1 (LATS1) as well as p-YAP127, were also lower in Col-NS, whereas phosphorylated MOB kinase activator 1 (p-MOB1) and p-YAP397 were not decreased but rather elevated, most notably in NS. MOB1 is an upstream cofactor of the Hippo kinase cascade, and phosphorylation of YAP at Ser127 and Ser397 governs its cytoplasmic retention and turnover; accordingly, the abundance of any single phospho-form does not, on its own, report net transcriptional activity. Because this mixed pattern across the cascade is not fully explained by the canonical MST–LATS–YAP model, we base our interpretation on the change in the total effector pool rather than on individual nodes. The functionally decisive observation is the pronounced reduction in total YAP and TAZ in Col-NS, indicating a lower abundance of the profibrotic effector pool available for nuclear signaling and suggesting that Col-NS limits excessive YAP/TAZ-associated mechanotransduction under wound healing conditions. This effect may be attributed to the aligned nanoscale architecture of Col-NS, which provides directional cell guidance and organized cytoskeletal remodeling, thereby reducing aberrant cellular tension and profibrotic mechanosignaling. In addition, the collagen coating may further enhance cell–matrix interactions in a physiologically relevant manner, supporting coordinated migration and matrix remodeling without triggering excessive fibrotic activation.

Quantitative analysis (Fig. 7I) showed that Col-NS reduced total YAP to ~0.49-fold and TAZ to ~0.65-fold relative to TCPS. Several upstream components were also lower in Col-NS (MST1 ~0.82, MST2 ~0.77, SAV1 ~0.81, and LATS1 ~0.81), as was p-YAP127 (~0.78), whereas p-MOB1 and p-YAP397 were elevated. Given this mixed pattern across the cascade, we interpret the data conservatively and focus on the total effector abundance rather than on any single phosphorylated species. Because the transcriptionally active nuclear fraction of YAP/TAZ cannot exceed the total cellular pool, the pronounced reduction in total YAP and TAZ lowers the ceiling on YAP/TAZ-driven transcription independently of the precise nuclear-to-cytoplasmic ratio and thus constrains the maximal mechanotransductive output attainable in Col-NS. This molecular change coincided with a strongly elongated, unidirectionally aligned cell morphology (Fig. 1K to M), a geometric state to which YAP/TAZ regulation is well-known to be sensitive, indicating that the nanotopography reshapes the cytoskeletal context in which these effectors operate. Moreover, because YAP/TAZ and TGF-β/SMAD signaling are mutually reinforcing during fibroblast activation, the concurrent suppression of the canonical SMAD axis (Fig. 7F and G) is consistent with a coordinated, rather than compensatory, down-regulation of the profibrotic transcriptional program. Together, these convergent molecular and morphological changes indicate a less profibrotic mechanotransductive state in Col-NS.

Collectively, these findings indicate that Col-NS exerts a dual regulatory effect on fibrosis-associated signaling pathways. By suppressing the TGF-β/SMAD axis, Col-NS reduces canonical profibrotic transcriptional activity related to fibroblast activation and ECM overproduction. Simultaneously, by reducing the total abundance of the YAP/TAZ effectors, Col-NS may attenuate abnormal mechanotransduction and matrix-stiffness-driven fibrotic responses. These molecular responses are consistent with the regenerative phenotype observed in the Col-NS group, suggesting that the collagen-coated nanotopographical scaffold does not merely serve as a passive wound covering material but actively provides biochemical and biophysical cues that guide tissue repair toward organized regeneration with minimized scar formation.

To dissect the individual roles of the 2 design elements, we compared flat, nanotopographical (NS), and collagen-coated (Col-NS) scaffolds across mechanical, cellular, and molecular assays. The aligned nanotopography served as the primary determinant of directional cell guidance: fibroblasts aligned along the 800-nm grooves at collagen concentrations of 1% or below, whereas this alignment was lost once the topography was masked at 2% collagen (Fig. 1I and J), indicating that contact guidance originates from the structural cue rather than from the coating. The nanotopography also accounted for a substantial portion of the improvement in tensile strength (from 17.65 to 23.06 MPa) and of the in vivo wound-closure benefit over Duoderm (Fig. 4). The collagen coating, by contrast, predominantly enhanced adhesion-dependent responses, providing the largest incremental gains in cell migration (Fig. 7B), tensile adhesion, proliferation, and osteogenic differentiation (Fig. 7C to E), consistent with the integrin-mediated (e.g., GFOGER–integrin) signaling supplied by type I collagen. Western blot analysis further revealed that suppression of TGF-β/SMAD and Hippo–YAP/TAZ signaling was already partially induced by NS and became most pronounced in Col-NS (Fig. 7F to I), suggesting that the nanotopography initiates the antifibrotic mechanotransductive response, which the collagen coating subsequently amplifies. Taken together, these comparisons indicate that the nanotopography chiefly governs structural guidance and mechanotransduction, whereas the collagen coating chiefly governs adhesion, proliferation, and biochemical signaling, and that their combination underlies the additive-to-synergistic regenerative performance of Col-NS.

Biomedical membrane patches are among the most extensively utilized biomaterial platforms in clinical regenerative medicine worldwide, owing to their ease of application, biocompatibility, and versatility across multiple tissue types. However, these conventional patches have long functioned as passive barriers providing structural protection without the intrinsic ability to initiate or orchestrate true tissue regeneration. In this study, we developed and translationally evaluated a collagen-coated PLGA nanotopographical scaffold (Col-NS) that integrates biochemical and biophysical ECM-mimetic cues. The principal strength of this work is its multilevel validation, spanning cellular and molecular analyses, normal and diabetic wound models, a calvarial defect model, and early human applications in skin and alveolar bone regeneration. These findings demonstrate that Col-NS can provide cell-instructive functions beyond passive wound coverage and support its development as a clinically relevant bioactive membrane platform. The successful clinical application of Col-NS represents a substantial advancement in regenerative medicine and scaffold-based therapies. This multifunctional platform combines the biochemical properties of type I collagen with the structural precision of nanoscale-aligned PLGA topography to address persistent challenges in skin and alveolar bone regeneration. This discussion explores the design rationale, underlying biological mechanisms, and translational relevance of the developed scaffold, highlighting its clinical advantages compared with current commercial solutions.

PLGA is one of the most widely used biodegradable polymers in clinical settings, valued for its excellent biocompatibility, tunable degradation kinetics, and mechanical processability [41]. Its degradation rate can be precisely tailored by altering the lactic-to-glycolic acid ratio, allowing alignment with various tissue regeneration timelines. Despite these advantages, PLGA exhibits limitations, including the generation of acidic degradation by-products, which may elicit localized inflammation, and poor intrinsic cell affinity, which impairs initial cell attachment and downstream signaling [42].

To overcome these limitations, incorporating surface nanotopography with a collagen coating has emerged as a promising strategy. The native ECM presents a hierarchical, anisotropic architecture spanning from individual collagen fibrils (~10 to 500 nm in diameter) to bundled collagen fibers at the micrometer scale [35]. The aligned 800-nm ridge-and-groove geometry used here was therefore not intended to replicate the diameter of a single collagen fibril; rather, it lies within this nano-to-microscale continuum, approximating the dimensional range of bundled fibrillar structures, and was selected primarily on the basis of previous studies, including our own, demonstrating that submicron aligned gratings robustly induce contact guidance, cytoskeletal and nuclear alignment, and focal adhesion formation in fibroblasts and osteogenic cells [36,37]. This topographical cue guides cellular behavior by modulating cytoskeletal organization, nuclear alignment, and focal adhesion formation [36]. This study reveals that such topographical surfaces substantially enhance cellular alignment and promote directional migration, which are essential for coordinated tissue regeneration, particularly in anisotropic tissues such as skin and bone.

However, nanotopographical PLGA surfaces alone cannot fully support cell adhesion or ECM remodeling due to their bioinert nature. Applying a type I collagen coating addresses this limitation by providing bioactive ligands (e.g., GFOGER motifs) that engage with integrin receptors on the cell surface, triggering signaling cascades that promote adhesion, proliferation, and differentiation [43]. Nevertheless, collagen coatings present several challenges, including susceptibility to enzymatic degradation, high production costs, and batch-to-batch variability [44]. In this study, the synergistic integration of PLGA nanotopography with collagen coating produced a scaffold that not only enhanced cell–substrate interactions but also maintained mechanical integrity and bioactivity over a clinically relevant period. Importantly, the integrated Col-NS platform was evaluated across both preclinical models and human applications involving skin and alveolar bone regeneration. This cross-tissue translational evaluation, together with the scaffold’s manufacturability, mechanical performance, and biological functionality, constitutes the principal contribution of the present study.

Notably, because nanotopography and collagen occupy the same physical surface, their contributions are concentration-dependent rather than simply additive: beyond an optimal loading, additional collagen masks the underlying relief and attenuates topographical guidance rather than enhancing the overall response. The 1% coating adopted here therefore represents a titrated balance that provides sufficient collagen-mediated biochemical support without occluding the 800-nm features. This underscores that in dual-cue ECM-mimetic scaffolds, biochemical functionalization must complement rather than override the structural cue, with the optimal response reflecting a balanced coexistence of both signals rather than a maximal collagen load.

The observed enhancement in cellular behavior on collagen-coated nanotopographical scaffolds is likely due to the combined influence of biochemical and biophysical cues. Collagen facilitates integrin-mediated adhesion and signaling, leading to focal adhesion kinase activation, cytoskeletal reorganization, and the downstream regulation of genes involved in proliferation and migration [45]. Simultaneously, the aligned nanotopography stimulates mechanosensitive pathways, including YAP/TAZ nuclear translocation, which influences lineage specification and matrix remodeling [46]. The interaction between these cues creates a dynamic microenvironment that supports tissue regeneration. In vitro findings from this study confirmed that fibroblasts and osteoblast-like cells cultured on collagen-coated nanotopography exhibited increased adhesion, proliferation, and osteogenic differentiation compared to those on flat or uncoated surfaces. Migration assays revealed accelerated wound closure, driven by enhanced lamellipodium formation and directionally persistent migration along the nanotopographical grooves. Mechanistically, the down-regulation of SMAD2/3 and SMAD4 within the TGF-β pathway suggests decreased profibrotic signaling, while the pronounced reduction in total YAP and TAZ in the Hippo pathway indicates a diminished pool of profibrotic mechanotransductive effectors, potentially mitigating scar tissue formation [47]. Together, these findings suggest that the scaffold not only promotes regeneration but also minimizes the risk of fibrotic encapsulation, a common complication in scaffold-based therapies. The proximate determinant of YAP/TAZ transcriptional activity is the nuclear-versus-cytoplasmic distribution of the effectors, which is governed by cell geometry and cytoskeletal tension. Our dataset characterizes the total effector pool and the cellular morphology rather than this partitioning directly; nevertheless, 2 features of the data speak to the overall profibrotic output. First, the size of the total YAP/TAZ pool sets an upper bound on nuclear-active signaling. Regardless of the partitioning ratio, the substantial reduction in total YAP and TAZ in Col-NS necessarily lowers the maximal achievable YAP/TAZ-driven transcription. Second, this reduction occurred in cells that were strongly elongated and contact guided (Fig. 1K to M), a geometry to which YAP/TAZ regulation is known to be sensitive, indicating that the nanotopography reshapes the cytoskeletal context in which these effectors operate. Because YAP/TAZ and TGF-β/SMAD signaling cooperate to drive fibroblast activation, their coordinated down-regulation on Col-NS is internally consistent and aligns with the regenerative, scar-minimized phenotype observed in vitro, in vivo, and clinically. We therefore interpret these findings at the level of a reduced profibrotic effector pool and a mechanically permissive cell geometry, without inferring a specific nuclear-to-cytoplasmic ratio. Building on this coherent picture, a spatially resolved evaluation of YAP/TAZ subcellular localization, quantifying its nuclear-to-cytoplasmic distribution in relation to single-cell cytoskeletal architecture, would add further mechanistic depth and represents a natural extension of the present work that would more directly link nanotopography-guided cytoskeletal organization to downstream mechanotransductive transcriptional activity.

The translational potential of the scaffold was validated through its performance across in vitro, in vivo, and clinical settings. In vitro studies demonstrated robust cell adhesion, migration, and differentiation, which were consistent with the enhanced regenerative outcomes observed in normal and diabetic wound models in vivo. The diabetic model, which represents a clinically challenging chronic wound environment characterized by impaired angiogenesis and prolonged inflammation, responded particularly well to treatment with the collagen-coated nanotopographical scaffold. This indicates that the scaffold provides structural support and molecular signals that modulate the chronic wound microenvironment toward a regenerative phenotype.

Furthermore, the efficacy of the scaffold in critical-sized bone defects confirms its application as a multifunctional material for hard- and soft-tissue regeneration. Quantitative μ-CT analysis revealed substantial increases in bone area and volume over an 8-week implantation period, while histological evaluation confirmed lamellar bone formation in close association with the scaffold matrix. The alignment of newly formed bone along the nanotopographical orientation further emphasizes the importance of topographical guidance in orchestrating regenerative processes.

Clinically, our scaffold platform demonstrated superior performance in human patients, in both cutaneous laser wound healing and alveolar ridge regeneration following tooth extraction. In the skin regeneration cohort, substantial improvements were observed in key parameters, including wound contraction, dermal thickness, skin roughness, and TEWL. No adverse events were reported, and patients exhibited rapid re-epithelialization with favorable cosmetic outcomes. In alveolar bone applications, all implants achieved successful osseointegration without signs of inflammation or peri-implant bone loss, even under functional loading. Histological analyses revealed well-vascularized new bone with minimal fibrotic infiltration, underscoring the clinical safety and osteoconductivity of Col-NS.

Compared to current commercial scaffolds and wound dressings, such as Duoderm, collagen matrices, or synthetic membranes, our platform offers several distinct advantages [48]. While existing materials may provide temporary wound coverage or passive structural support, they often lack the integrated biochemical and biophysical sophistication required for orchestrated tissue regeneration. For example, Duoderm dressings primarily function as moisture-retaining barriers and lack cell-instructive properties. In contrast, our scaffold actively engages cellular processes through its ECM-mimetic nanotopography and bioactive collagen coating [49].

Furthermore, commercial collagen membranes used for GBR often degrade too rapidly or lack sufficient mechanical strength for space maintenance, particularly in vertical bone augmentation. Our scaffold addresses this through enhanced tensile strength (up to 29.86 MPa) and adhesion properties, enabling robust handling and implantation in open and closed membrane techniques. This study provides an early clinical demonstration of an ECM-mimetic nanostructured scaffold engineered with collagen coating and aligned nanotopography for both skin and bone regeneration in humans. These findings support the feasibility of translating high-fidelity biomimetic scaffold design, previously largely confined to in vitro or preclinical research, into clinical practice.

The dual-functional design of the collagen-coated PLGA nanotopographical scaffold suggests potential for broader applicability beyond the scope of this study. The platform can be readily adapted for other clinical indications, including chronic ulcers, surgical reconstruction, or orthopedic repair, particularly in patients with systemic comorbidities such as diabetes, vascular disease, or advanced age. Its mechanical profile, tunable degradation, and modular bioactivity also provide a foundation for integrating controlled drug release, gene delivery, or stem cell seeding to further enhance regenerative potential.

## Conclusion

In conclusion, we present the design, development, and clinical validation of an active tissue-regenerative biomedical membrane patch, introducing a concept that extends beyond conventional passive functionality. Through the integration of biomimetic nanotopographical cues and collagen-based biochemical signals within a collagen-coated PLGA nanotopographical scaffold (Col-NS), we established a next-generation platform capable of directly stimulating cellular and molecular processes that underlie true tissue regeneration. Based on comprehensive preclinical and clinical evaluations, Col-NS demonstrated substantial therapeutic efficacy in both human skin and alveolar bone regeneration. Clinically, the scaffold markedly improved wound healing parameters including contraction, dermal recovery, and skin barrier function without any adverse effects. In alveolar bone applications, all patients exhibited stable implant integration, preserved graft height, and healthy peri-implant mucosa, resulting in a 100% implant success rate. Histological analyses confirmed mature lamellar bone formation and excellent biocompatibility with no inflammatory response. Quantitative data indicated over 90% improvement in skin roughness, TEWL, and wound area reduction within 7 d, highlighting accelerated tissue repair. Mechanistic investigations revealed that Col-NS effectively modulates key regenerative signaling pathways such as TGF-β and Hippo, thereby promoting functional tissue regeneration while minimizing fibrotic and scar-forming responses. Collectively, this study provides, to our knowledge, one of the first clinical validations of an ECM-mimetic, collagen-coated PLGA aligned nanotopographical scaffold as a safe and effective regenerative platform across both soft and hard tissues. This work may help expand the role of biomedical membrane patches from passive wound and tissue coverings toward active, regenerative therapeutics and represents a meaningful step in the ongoing development of clinically translatable biomaterials.

## Data Availability

The datasets used and/or analyzed during the current study are available from the corresponding authors upon reasonable request.
