# Supplementary material for: Regenerative Nano-scaffolds for Tissue Engineering: From Bench to Clinical Translation
Source: Biomater Res. 2026 Aug 31;30:0414. doi: 10.34133/bmr.0414 (PMC13527211; doi:10.34133/bmr.0414)
Supplement: Supplementary file 1 [file bmr.0414.f1.docx]

Supplementary Materials

Title: Regenerative Nano-Scaffolds for Tissue Engineering: from Bench to Clinical Translation

Woochan Kim^1,2,3,†^, Sangbae Park^4,5,6,†^, Sang Won Beom^7^, Selin Choi^7^, Hye-Won Yang^7^, Dream Kim^1,2,3^, Shinyull Lee^1,2,3^, Harshita Sharma^1,2,3^, Chaeyeon Park^1,2,3^, Won-Pyo Lee^8,*^, Jangho Kim^1,2,3,7,*^

^1^ Department of Convergence Biosystems Engineering, Chonnam National University; Gwangju 61186, Republic of Korea.

^2^ Department of Rural and Biosystems Engineering, Chonnam National University; Gwangju 61186, Republic of Korea.

^3^ Interdisciplinary Program in IT-Bio Convergence System, Chonnam National University; Gwangju 61186, Republic of Korea.

^4^ Department of Biosystems Engineering, Seoul National University; Seoul 08826, Republic of Korea.

^5^ Integrated Major in Global Smart Farm, College of Agriculture and Life Sciences, Seoul National University; Seoul 08826, Republic of Korea.

^6^ Research Institute for Agriculture and Life Sciences, Seoul National University; Seoul, 08826, Republic of Korea.

^7^ Institute of Nano-Stem Cells Therapeutics, NANOBIOSYSTEM Co., Ltd; Gwangju 61003, Republic of Korea.

^8^ Department of Periodontology, School of Dentistry, Chosun University; Gwangju 61452, Republic of Korea.

* Corresponding author email: Jangho Kim (rain2000@jnu.ac.kr) and Won-Pyo Lee (wplee8@chosun.ac.kr).

^†^ Woochan Kim and Sangbae Park contributed equally to this work.

Keywords: Nanotopographical scaffolds, Collagen coating, ECM-mimetic, Clinical translation, Skin regeneration, Bone regenration, Aveloar bone regeneration

Fig. S1. Concentration-dependent fibroblast morphology and alignment on Col-NS. (A) Representative FE-SEM images and (B) F-actin (phalloidin, red) fluorescence images of NIH-3T3 fibroblasts cultured for 24 h on scaffolds coated with 0.1%, 0.5%, 1%, and 2% collagen. Double-headed arrows indicate the nanopattern direction.

Fig. S2. Vinculin-based focal adhesion analysis on Col-NS. (A) Anti-vinculin (green) images of fibroblasts on 0.1%, 0.5%, 1%, and 2% collagen (scale bar: 100 μm). (B) Vinculin orientation relative to the nanopattern (-90° to 90°). (C) Cell aspect ratio and (D) focal adhesion area. Data are presented as mean ± SD. *p < 0.05, **p < 0.01, ***p < 0.001, determined via one-way ANOVA followed by Tukey’s HSD post hoc test.

**Fig. S3. Observations of the NS explanted from regenerated human alveolar tissue. (A)** Macroscopic assessment showing the explanted NS retained mechanical integrity and gross geometry with no evidence of bulk degradation or structural collapse. **(B)** High-resolution SEM iamge of the explanted NS demonstrating complete preservation of the engineered ridge–groove nanotopography. **(C)** Histomorphology of tissue adherent to the NS surface, demonstrating anisotropic organization of cells and ECM with parallel alignment and increased packing density along the nanotopography direction.
